# The search for new amphiphiles: synthesis of a modular, high-throughput library

**DOI:** 10.3762/bjoc.10.163

**Published:** 2014-07-10

**Authors:** George C Feast, Thomas Lepitre, Xavier Mulet, Charlotte E Conn, Oliver E Hutt, G Paul Savage, Calum J Drummond

**Affiliations:** 1 CSIRO Materials Science and Engineering, Bag 10, Clayton South MDC, VIC 3169, Australia; 2 School of Applied Sciences, College of Science, Engineering and Health, RMIT University, GPO Box 2476, Melbourne, VIC 3001, Australia

**Keywords:** amphiphiles, carbohydrates, click chemistry, high throughput, library synthesis

## Abstract

Amphiphilic compounds are used in a variety of applications due to their lyotropic liquid-crystalline phase formation, however only a limited number of compounds, in a potentially limitless field, are currently in use. A library of organic amphiphilic compounds was synthesised consisting of glucose, galactose, lactose, xylose and mannose head groups and double and triple-chain hydrophobic tails. A modular, high-throughput approach was developed, whereby head and tail components were conjugated using the copper-catalysed azide–alkyne cycloaddition (CuAAC) reaction. The tails were synthesised from two core alkyne-tethered intermediates, which were subsequently functionalised with hydrocarbon chains varying in length and degree of unsaturation and branching, while the five sugar head groups were selected with ranging substitution patterns and anomeric linkages. A library of 80 amphiphiles was subsequently produced, using a 24-vial array, with the majority formed in very good to excellent yields. A preliminary assessment of the liquid-crystalline phase behaviour is also presented.

## Introduction

**Figure 1:**
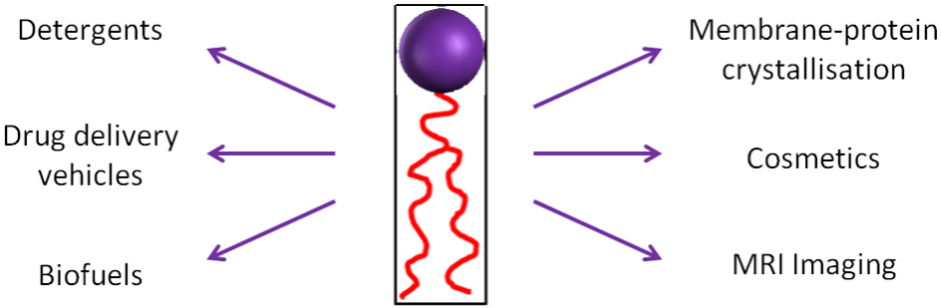
Examples of amphiphile applications.

Amphiphilic compounds contain a hydrophilic polar head group and a hydrophobic non-polar side chain. Upon addition of water, these amphiphiles may self-assemble into lyotropic phases that have a variety of uses, from simple household detergents and cleaning products, to biomedical applications including MRI imaging agents [1–3], membrane-protein crystallisation media [4–6] and solubilising bioactive food additives (Figure 1) [7]. Furthermore, recent research has centred on the use of amphiphile nanoparticles for drug-delivery applications [8–11].

Each of these applications requires a specific, stable liquid-crystalline phase. The phase behaviour can have a significant impact on performance for the end application with, for example, drug-release rates known to vary depending on the geometrical characteristics of the lyotropic phase [12]. Increasing the lattice parameter of the liquid-crystalline phase may also facilitate the uptake of larger bioactive molecules [4].

At present, only a small selection of amphiphiles is used in the aforementioned applications. Research into the design, synthesis, and material characterisation of new amphiphiles has been underexplored, generally due to technical difficulties in the synthesis and handling of such compounds [13]. Thus, a new method combining high-throughput synthesis and liquid-crystalline phase characterisation may open new territory in the field of amphiphile discovery.

Furthermore, whilst the degree of lipophilicity, the polarity of the molecule, and the volume and shape of the molecule have all been attributed to controlling phase behaviour, the understanding of the interplay and relative contribution of these factors is still evolving. Therefore, in addition to discovering new amphiphilic species, the synthesis of a combinatorial library of amphiphilic compounds would allow the factors that drive self-assembly and liquid-crystalline phase formation to be analysed. Such studies are critical to the further development and design of new amphiphiles tailored for a specific application, and to deepen the understanding of how molecular structure influences the characteristics of self-assembly.

Previous amphiphile libraries have been prepared using a thiol–yne reaction [14] and an in situ hydrazone formation between aldehyde tails and hydrazide head groups [15] in order to study gene delivery. Many other fields have utilised the copper-catalysed azide–alkyne cycloaddition (CuAAC) ‘click’ reaction [16–17] to generate libraries of compounds, including enzyme inhibitors [18–20], catalysis ligands [21–23] and metal frameworks [24–25]. We have recently demonstrated that a library of amphiphiles, with ammonium head groups and single-chain saturated tails, can be synthesised in a combinatorial approach, using this chemistry [26]. Amphiphiles and self-assembled nanoparticles have been synthesised using CuAAC chemistry previously [27–29], however to our knowledge, this was the first amphiphile library synthesised in this manner.

**Figure 2:**
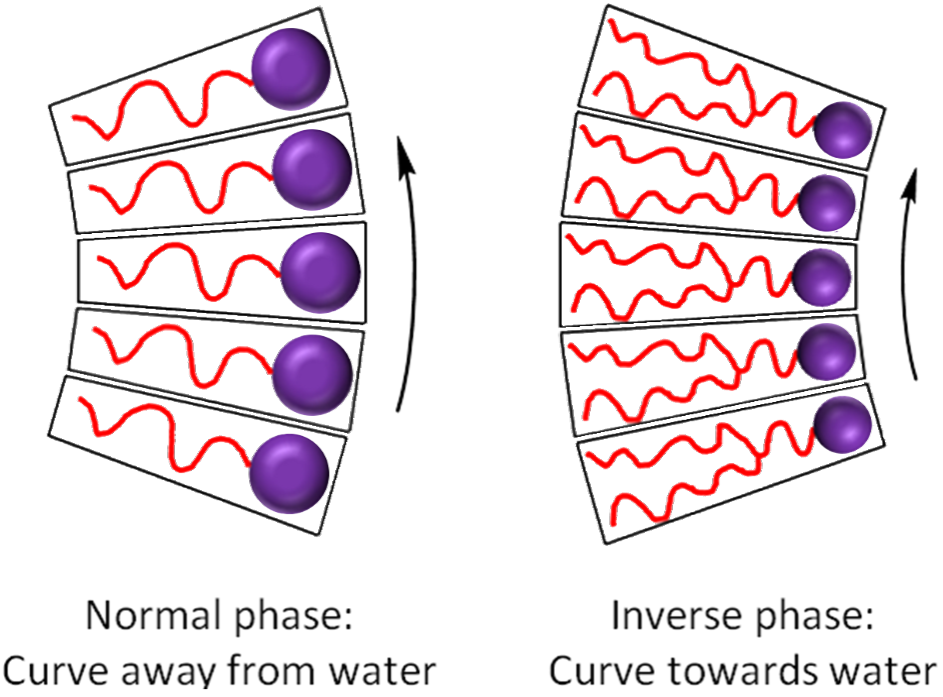
Upon self-assembly, amphiphiles pack and curve away from (normal phase) or towards (inverse phase) water.

In a follow-up study, this protocol was applied to produce amphiphiles with single-chained saturated, unsaturated, and branched tails with sugar head groups [30]. This library of amphiphiles was analysed by high-throughput synchrotron small-angle X-ray scattering (SSAXS), to determine the liquid-crystalline phases of the individual compounds. These amphiphiles were found to form normal phases that have interfaces that curve away from water (Figure 2). For the majority of biomedical applications, inverse phase-forming amphiphiles (with interfaces that curve towards the aqueous domain) are desirable as they maintain the same lyotropic structure upon dilution [8].

Inverse phases typically form from amphiphiles with multiple hydrocarbon-chain tails. Therefore, to tune our amphiphiles towards inverse phase formation, we undertook the synthesis of double and triple-chain, alkyne-tethered tails, and subsequently used these to synthesise a library of multi-chained amphiphiles in a high-throughput manner.

## Results and Discussion

In order to synthesise double and triple-chain tails with a variety of chain types, a synthetic route was required that enabled a late-stage, modular addition of the hydrocarbon chains; the products of which could be taken straight into the high-throughput CuAAC reaction.

**Scheme 1:**
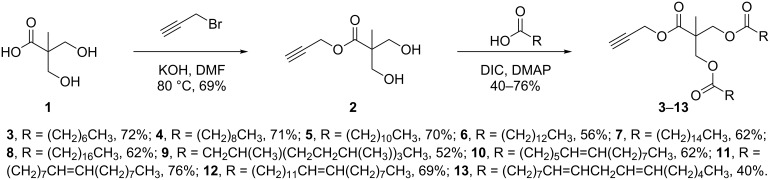
Synthesis of double-chain, alkyne-tethered tails.

To this end, diol **2** was synthesised by esterification of commercially available 3-hydroxy-2-(hydroxymethyl)-2-methylpropanoic acid (**1**), according to the procedure of Whittaker et al. (Scheme 1) [31]. Diol **2** was subsequently acylated twice using a variety of long-chain carboxylic acids, and diisopropylcarbodiimide (DIC) as a coupling agent, to afford double-chain tails **3**–**13** in predominantly good yields. This method avoided the need to protect the diol prior to acylation [32].

**Scheme 2:**
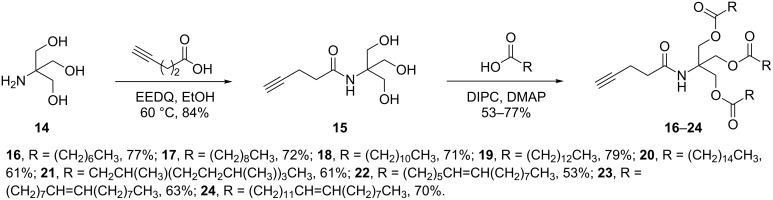
Synthesis of triple-chain, alkyne-tethered tails.

For the triple-chained tails, triol **15** was synthesised from tris(hydroxymethyl)methylamine (TRIS) and 4-pentynoic acid using ethoxycarbonyl-2-ethoxy-1,2-dihydroquinoline (EEDQ), following a similar method to Pucci et al. (Scheme 2) [33]. TRIS has been used previously for the synthesis of amphiphilic drug-delivery vehicles, with advantages including its inexpensive availability and non-toxic nature [34–35].

Treatment of core molecule **15** with 3.5 equivalents of various fatty acids, using a similar coupling procedure as for the double-chain tails, gave alkynes **16–24** in moderate to good yields. Lower yields were attributed to steric hindrance of the second and third esterifications, as well as the presence of mono- and diesters making the purification more difficult. 4-Pentynoic acid was used rather than 3-butynoic acid due to the relative price and availability, as well as placing the alkyne a further methylene unit from the very bulky tertiary centre, which may have hindered the CuAAC reaction.

Using the above methods, a total of twenty tails were synthesised (11 double and 9 triple-chained, Table 1). These consisted of saturated chains with systematic ethylene increases between (CH_2_)_6_CH_3_ (abbreviated to C7) and (CH_2_)_16_CH_3_, as well as branched (phytanic), monounsaturated (palmitoleic, oleic and erucic) and polyunsaturated (linoleic) chains, which have been shown to promote the formation of inverse hexagonal and/or inverse bicontinuous cubic lyotropic phases in related systems [36–38].

**Table 1:** Double and triple-chain alkyne-tethered tails synthesised.^a^

	Core	**2**		**15**	
Fatty acid	Abbrev.^b^	Compound	Yield (%)	Compound	Yield (%)

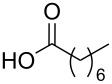	C7	**3**	72	**16**	77
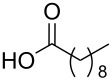	C9	**4**	71	**17**	72
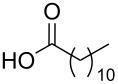	C11	**5**	70	**18**	79
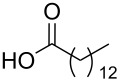	C13	**6**	56	**19**	74
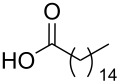	C15	**7**	62	**20**	61
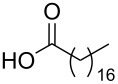	C17	**8**	62	**–**	–
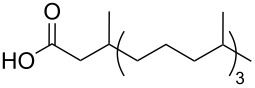	Phyt	**9**	52	**21**	61
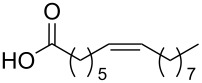	Palm	**10**	62	**22**	53
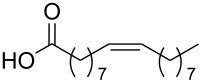	Ole	**11**	76	**23**	63
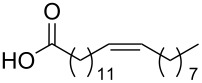	Eruc	**12**	69	**24**	70
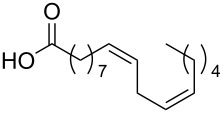	Lin	**13**	40	–	–

^a^A dash indicates those not synthesised. ^b^Phyt = phytanic, Palm = palmitoleic, Ole = oleic, Eruc = erucic and Lin = linoleic.

A summary of the tail syntheses are presented in Table 1. The largest saturated tail (C17) was not synthesised as a triple chain due to the poor solubility of the product, while linoleic tails were only synthesised for double chains as the volume of a triple-chain tail would be unfavourable for lyotropic phase formation. The yields for each chain type are fairly consistent between double and triple-chain analogues, highlighting the practicality of using this modular approach. These compounds were able to go directly into the high-throughput CuAAC reactions to synthesise double and triple-chain amphiphiles.

With tails in hand, attention was turned to the hydrophilic amphiphile head groups. Sugar head groups were selected for this library because sugars are popular drug targets; however, such compounds often suffer from poor bioavailability [39]. By loading such drugs as amphiphilic assemblies, sugar recognition and subsequent excretion should be reduced [40–42]. Sugar-based amphiphiles are often termed glycolipids and are also of interest for their use as ‘green’ surfactants in household cleaning products [43]. Previous work in this field has been extensively reviewed [44–50]. Azido-sugars are known to react well under CuAAC reaction conditions [51–52] and have recently been used in the synthesis of a glycodendrimer library [53], as well as in our previous work on single-chain sugar amphiphiles [30]. Hence, a diverse sugar screen would provide a wealth of data on the appropriate characteristics for a self-assembled sugar-based amphiphilic drug.

**Figure 3:**
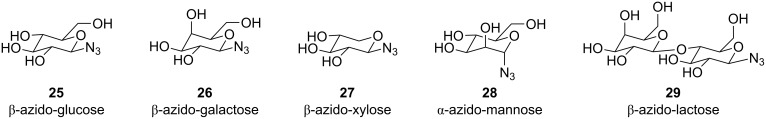
Azido-sugar head groups used in library.

Five sugars were chosen for this library: glucose, galactose, lactose, xylose and mannose. Glucose and galactose amphiphiles have been demonstrated to possess slightly different lyotropic phase behaviour, despite the relatively small structural difference (epimers) between the two monosaccharides. Xylose does not bear the 6-position side-chain alcohol so it takes up a significantly smaller volume. Lactose is a disaccharide so it has a much larger head-group volume. The four azido-sugars associated with these sugars are linked through a β-anomeric linkage, therefore an α-linked mannose was also selected for comparison (Figure 3).

**Scheme 3:**
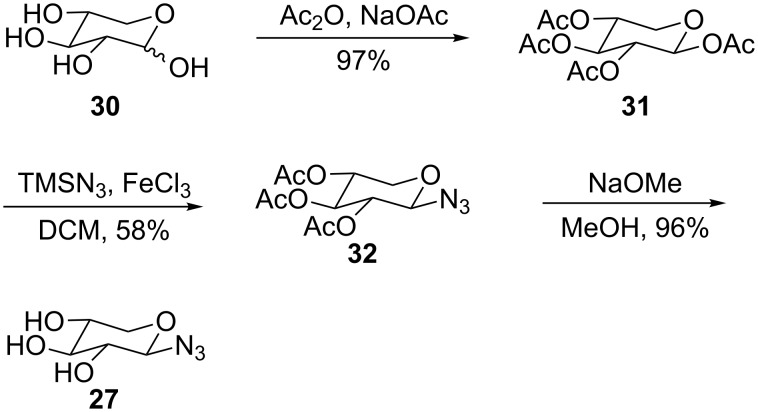
Synthesis of azido-xylose.

These azido-sugars were commercially available with the exception of azido-xylose **27**, which was synthesised in three steps from the parent sugar using trimethylsilylazide and iron(III) chloride (Scheme 3) [54]. The β-configuration of the azide was confirmed by comparison of coupling constants with literature data [55].

**Figure 4:**
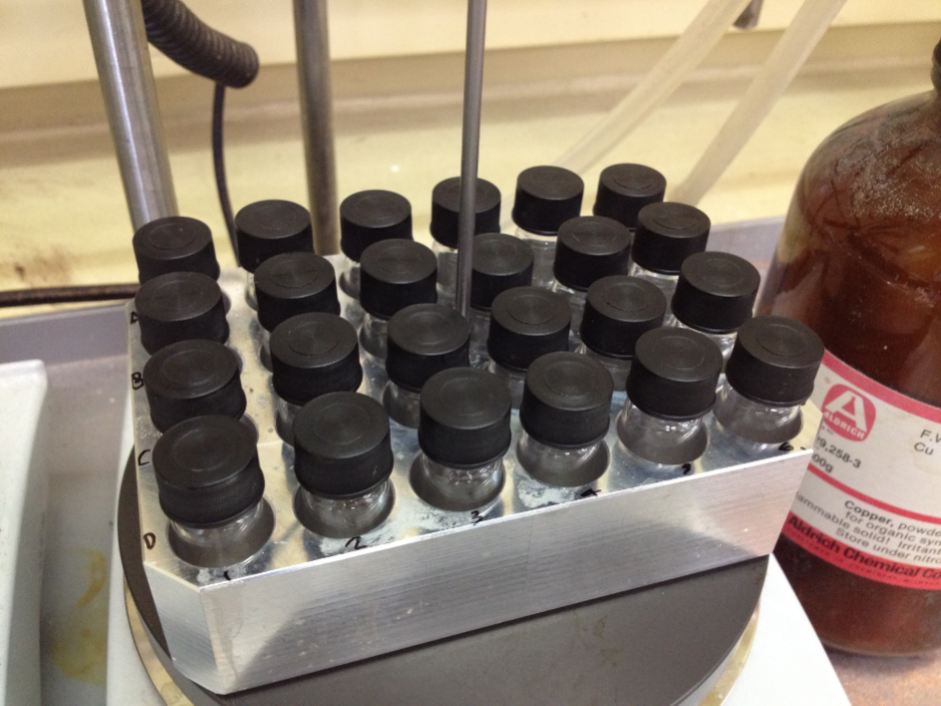
24-vial array set up.

**Figure 5:**
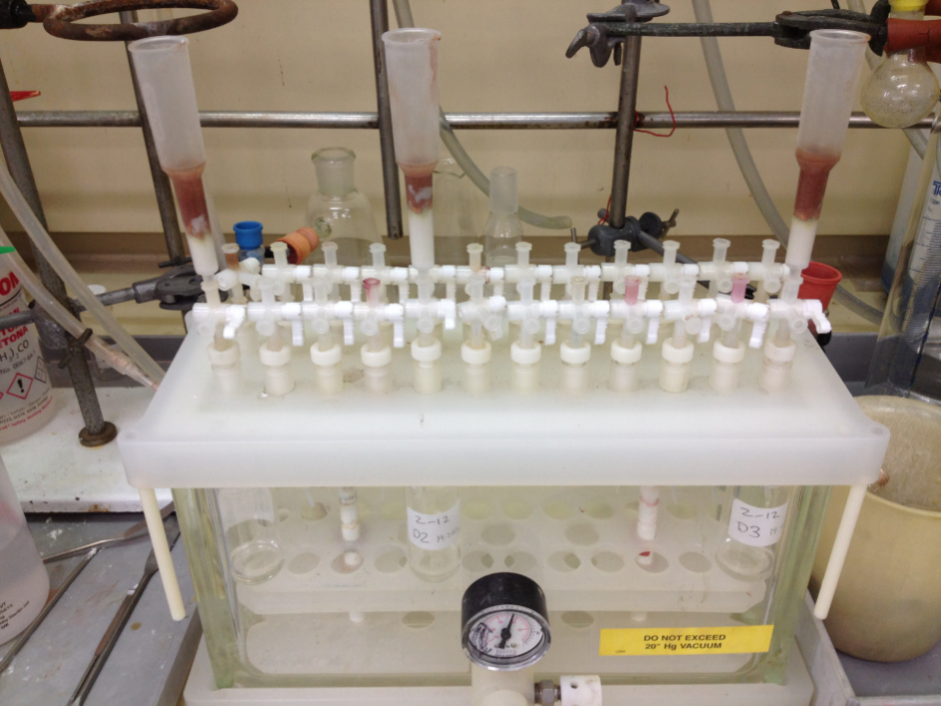
Multi-tap vacuum chamber for high-throughput filtering.

With both head groups and tails in hand, high-throughput CuAAC reactions were employed to synthesise amphiphiles using a 24-vial array. Azido-sugars were dissolved in a mixture of *tert*-butanol and water in 2 mL glass vials, followed by the addition of the alkyne tails (Figure 4). These were heated to 40 °C to dissolve the alkyne, before the addition of copper powder. After stirring for 24 h (48 h for mannose compounds), the reaction mixtures were filtered into vials using a multi-tap vacuum chamber (Figure 5) and the solutions evaporated to dryness simultaneously, using a vacuum centrifuge, to yield 24 amphiphiles.

This process was repeated to yield 80 novel amphiphiles, 46 with double-chain tails and 34 with triple-chain tails. The yields for the double-chain amphiphiles are presented in Table 2.

**Table 2:** Synthesis of double-chained amphiphiles.^a^

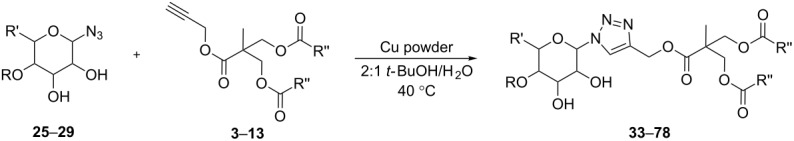										

Sugar	Glucose		Galactose		Xylose		Mannose		Lactose	

Tail (R'')	Comp.	Yield (%)	Comp.	Yield (%)	Comp.	Yield (%)	Comp.	Yield (%)	Comp.	Yield (%)

C7	**33**	92	**42**	90	**53**	93	**64**	87	**70**	89
C9	**34**	90	**43**	86	**54**	88	**65**	85	**71**	92
C11	**35**	89	**44**	88	**55**	86	**66**	68	**72**	94
C13	**36**	85	**45**	88	**56**	92	x		**73**	88
C15	–		**46**	80	**57**	94	–		–	
C17	–		**47**	60	**58**	65	–		–	
Phyt	**37**	88	**48**	91	**59**	87	**67**	82	**74**	78
Palm	**38**	90	**49**	87	**60**	88	**68**	82	**75**	80
Ole	**39**	85	**50**	78	**61**	81	**69**	76	**76**	74
Eruc	**40**	76	**51**	86	**62**	70	x		**77**	84
Lin	**41**	82	**52**	96	**63**	96	x		**78**	92

^a^Dashes indicate those not attempted, x indicates those which did not reach completion. Tail abbreviations are found in Table 1.

Almost all compounds were synthesised in very good to excellent yields. The yields were consistent both in terms of the sugar head group and the length and nature of the tail, emphasising the synthetic utility of this route towards amphiphile synthesis. Only for the longest of the saturated chains (C17) was a moderate yield observed; a ^1^H NMR sample of the galactose amphiphile **47** proved difficult to prepare (due to poor solubility) and the presence of unreacted azido-sugar was prevalent in the spectrum. The lower yield was therefore attributed to the poor solubility of the tails in the butanol/water solvent combination and thus the longest saturated chain (C15 and C17) amphiphiles were only synthesised from galactose and xylose.

Comparable yields were observed for the β-linked head groups for the short-chain saturated tails and the phytanic, palmitoleic and oleic tails. However the mannose reactions of chains longer than C11 and oleic were unable to reach completion, which was attributed to the α-linkage of the azide at the anomeric centre, creating steric hindrance for the [3 + 2] cycloaddition.

The yields for triple-chain amphiphiles are presented in Table 3. For the three saturated chains (C7, C9 and C11), excellent yields were obtained for most head groups, with good yields observed for the sterically-demanding α-linked mannose. For the phytanyl-chained amphiphiles moderate to very good yields were observed. This decrease in yield, attributed to increased steric interactions, was also observed for the longer chain erucic compounds, although these may also have suffered from poor solvation. ^1^H NMR of an example phytanyl compound **96** showed the presence of azido-xylose starting material, confirming that the reaction did not proceed to completion. Attempts to synthesise amphiphiles using the C13 (**19**) and C15 (**20**) triple-chain tails failed, as these compounds did not dissolve readily under the library synthesis reactions conditions.

**Table 3:** Synthesis of triple-chained amphiphiles.^a^

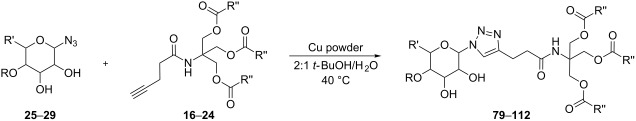										

Sugar	Glucose		Galactose		Xylose		Mannose		Lactose	
Tail (R'')	Comp.	Yield (%)	Comp.	Yield (%)	Comp.	Yield (%)	Comp.	Yield (%)	Comp.	Yield (%)

C7	**79**	89	**86**	95	**93**	96	**100**	71	**106**	88
C9	**80**	98	**87**	95	**94**	99	**101**	61	**107**	96
C11	**81**	89	**88**	93	**95**	85	**102**	73	**108**	92
Phyt	**82**	79	**89**	69	**96**	63	**103**	52	**109**	68
Palm	**83**	89	**90**	81	**97**	89	**104**	96	**110**	79
Ole	**84**	91	**91**	89	**98**	78	**105**	82	**111**	69
Eruc	**85**	60	**92**	50	**99**	69	x		**112**	53

^a^x indicates those which did not reach completion. Tail abbreviations are found in Table 1.

When comparing double and triple-chain amphiphiles, the results are similar. Excellent yields are observed for all C7 and C9-chained tails, with the exception of triple-chained α-linked mannose. A noticeable difference is observed, however, when comparing the yields of the phytanyl-chained amphiphiles; the triple chains exhibit yields 10–30% lower than for their double-chain analogues. It is reasonable to attribute this decrease to the increased steric demands of the branched chains. Both palmitoleic and oleic chain yields are reasonably consistent between double and triple, while a reduction in yield is observed for the very long erucic chains for the triple analogues.

The purity of the compounds was determined by ^13^C NMR and mass spectrometry. Since individual NMR analysis was impractical for such a large library, a random selection of 15 amphiphiles underwent ^13^C NMR spectroscopy representing three of each type of sugar and covering a range of double and triple tails. These spectra were found mostly to be of very good quality, with only minor amounts of starting material present in some samples (see data and spectra in Supporting Information File 1, S22–S32). Only one sample (triple-chain lactose amphiphile **106**) was found to contain significant amounts of tail starting material. It is postulated that a longer reaction time would have allowed complete conversion of the two bulky reactants into product.

All amphiphiles were subjected to MALDI–TOF mass spectrometry to determine product formation, and of the 80 amphiphiles in the library, only 3 (4%) did not generate the desired mass unit (Supporting Information File 1, Table S1).

In order to determine the purity of the compounds with regard to residual copper, inductively coupled plasma mass spectrometry (ICP–MS) was performed on three random samples (since a large amount of material is required for this technique). The results are presented in Table 4.

**Table 4:** Copper content analysis of amphiphiles.^a^

Amphiphile		^63^Cu content (ppm)

Galactose−2 × Phyt	**48**	20
Glucose−3 × C7	**79**	550
Lactose−3 × C9	**107**	80

^a^Tail abbreviations are found in Table 1.

All three compounds had copper contents <0.06% and, when combined with the ^13^C NMR and MALDI–TOF data, we were satisfied the compounds were pure enough for subsequent lyotropic phase data to be of significance in the context of a screen.

**Figure 6:**
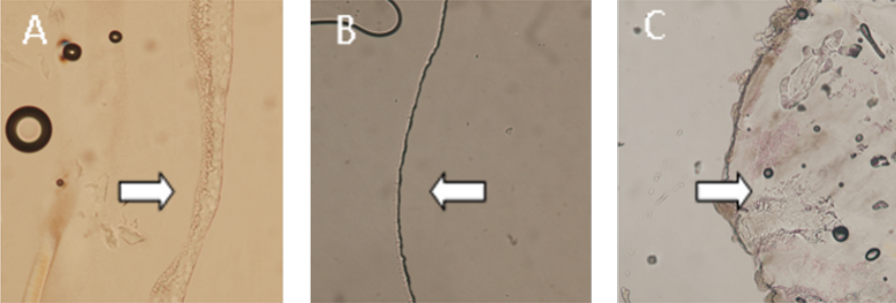
Cross-polarised microscopy of (A) glucose 2 × C7, **33**, (B) xylose 2 × C7, **53**, and (C) lactose 2 × C7, **70** amphiphiles under excess water conditions at 25 °C. The arrows indicate the phase at the water–amphiphile boundary.

The amphiphiles produced in this library are tailored to preferentially form inverse phases due to the large chain volume of the double and triple chains (Figure 2). Variations in the degree of curvature upon amphiphile packing lead to different individual phases [56–58]. As the propensity for curvature increases, the phase transitions are often lamellar → cubic → hexagonal → micellar [36]. An assessment of the phase behaviour of some amphiphiles was undertaken using cross-polarised microscopy. Each type of phase exhibits a distinct optical texture under the influence of polarised light [59]. Three compounds were chosen with different head groups (glucose, xylose and lactose) but the same double-chain tail (C7). The samples were placed between a microscope slide and a cover slip before flooding with water. The phase at the water–amphiphile interface was then observed (Figure 6).

**Figure 7:**
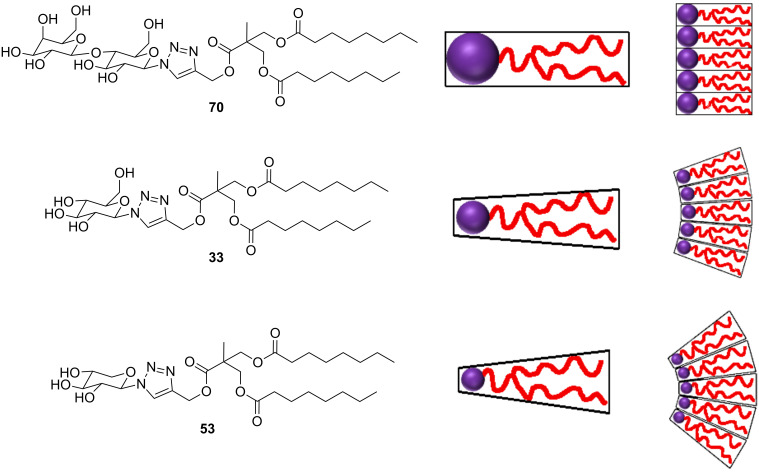
Differences in head group volume lead to differences in the curvature (and thus liquid-crystalline phase) of the self-assembled material.

The images A and B show an isotropic phase at the water–amphiphile boundary (confirmed by a dark image when viewed with crossed polarisers) indicating the presence of either a cubic or micellar phase. By contrast the lactose amphiphile **70** (Figure 7) shows a birefringent texture corresponding to a lamellar phase. Preliminary small-angle X-ray scattering data for these amphiphiles at high water concentrations match these assignments, with glucose amphiphile **33** exhibiting a gyroid inverse cubic phase, and xylose amphiphile **53** exhibiting an inverse micellar phase, at 25 °C. This clearly follows the expected pattern of increasing curvature with decreasing head group size (Figure 7). A disaccharide, such as lactose, has a large volume; however, in this amphiphilic form, the volume of the head group is matched by the volume of the double hydrocarbon chains and therefore, in water, the compound assembles into a lamellar phase. Upon reducing the head group size to glucose, we obtain a wedge-like amphiphile that curves towards water to give a material that self-assembles into an inverse cubic phase. Decreasing the head group size further to xylose, results in the formation of a highly curved inverse micellar phase.

When we combine these results with those from our single-chain amphiphile library (that form normal phases) [30], we are able to achieve lyotropic phases ranging across the structural landscape of normal to inverse micelles. Upon completion of the comprehensive phase behaviour analysis of this second library, using high-throughput small-angle X-ray scattering (SAXS) at the Australian Synchrotron, we predict that amphiphiles will be found of various inverse phases, thus creating a template from which novel amphiphiles can be synthetically designed for a specific end application.

## Conclusion

A library of double and triple-chained amphiphiles was synthesised using a modular, high-throughput approach. The compounds have systematic variations in chain length, splay and head-group size in order to gain knowledge of the relationship between molecular structure, and the structure of the self-assembled form of the amphiphile. The core molecules synthesised in this modular approach are highly adaptable and could be used to make double and triple-chained tails with differing chains on each arm. A preliminary investigation of three amphiphiles with various sized sugar head groups confirms that by decreasing the volume of the head group we are increasing the curvature at the amphiphile–water interface and thus move towards desired inverse phase formation.

## Experimental

**General procedure for the synthesis of amphiphiles.** To each of 24 glass vials (18 mm × 45 mm) in a 4 × 6 array aluminium reaction block, was added a solution of azido-sugar (1.0 equiv, ca. 15 mg) in 2:1 *t*-BuOH/water (1.5 mL). Alkyne (1.0 equiv) was added and the reaction block heated with stirring, to 40 °C. After dissolution, copper powder (ca. 150 mg) was added and the reaction stirred for 24–48 h. The reaction mixture was cooled, diluted with ethanol (2 mL) and filtered through Celite^®^ into 24 glass vials (25 mm × 75 mm). Concentration in vacuo on a Genevac EZ-2, followed by vacuum oven drying (50 °C, 3 h), afforded the amphiphile products.

**Cross-polarised microscopy.** A small amount of neat amphiphile was placed onto a microscope slide and a cover slip placed over gently pressing to form a watertight layer of amphiphile prior to hydration of the material. The microscope slide was placed into a Linkam PE94 hot stage (Linkam Scientific Instruments Ltd.; Surrey, England) and a water drop added at the side of the cover slip to flood the sample and create a concentration gradient as water was absorbed. The interaction of water and the amphiphile was observed with a Nikon Eclipse 80i inverted microscope (Coherent Scientific, Melbourne) without and with an analyser. Images were captured with a Nikon DS-Fi1 camera (Coherent Scientific, Melbourne).

## Supporting Information

File 1 Experimental procedures, chemical characterisation data (including ^13^C NMR spectra) and preliminary SAXS analysis.
