# Supplementary material for: The search for new amphiphiles: synthesis of a modular, high-throughput library
Source: Beilstein J Org Chem. 2014 Jul 10;10:1578–88. doi: 10.3762/bjoc.10.163 (PMC4142986; doi:10.3762/bjoc.10.163)
Supplement: File 1 — Experimental procedures, chemical characterisation data (including 13C NMR spectra) and preliminary SAXS analysis. [file Beilstein_J_Org_Chem-10-1578-s001.pdf]

Supporting Information
for
The search for new amphiphiles: synthesis of a modular, high-throughput library

George C. Feast[1], Thomas Lepitre[1], Xavier Mulet[1], Charlotte E. Conn[1], Oliver E. Hutt[1], G. Paul Savage*[1], Calum J. Drummond*[1,2]

Address: [1]CSIRO Materials Science and Engineering, Bag 10, Clayton South MDC, VIC 3169, Australia and [2]Current address: School of Applied Sciences, College of Science, Engineering and Health, RMIT University, GPO Box 2476, Melbourne, VIC 3001, Australia.

Email: G. Paul Savage*- paul.savage@csiro.au; Calum J. Drummond*- calum.drummond@csiro.au

*Corresponding author

Experimental procedures, chemical characterisation data (including ^{13}C NMR spectra) and preliminary SAXS analysis

Contents

S2 General experimental

S3 Synthesis of double-chain tails

S11 Synthesis of triple-chain tails

S18 Synthesis of sugar head groups

S20 Amphiphile synthesis and characterisation

S25 ^{13}C NMR spectra

S32 Preliminary SAXS analysis

S33 References

General experimental

All solvents used were HPLC grade and all chemicals were purchased from Sigma-Aldrich.

Nuclear magnetic resonance (NMR) spectra were recorded on a Bruker AV400 spectrometer. ^{1}H NMR and ^{13}C NMR were recorded at 400 MHz and 100 MHz, respectively. ^{1}H NMR chemical shifts are reported in ppm with the internal chloroform signal at 7.26 ppm. The data are reported as integration, s = singlet, d = doublet, t = triplet, q = quartet, non = nonet, m = multiplet, br = broad, app. = apparent, *J* = coupling constant(s) in Hz. ^{13}C NMR chemical shifts are reported in ppm with the internal chloroform signal at 77.0 ppm.

Amphiphile ^{13}C NMR spectra were recorded using a Bruker Avance III HD 600 MHz spectrometer with a TCI cryoprobe. The experimental parameters were: 32894.738 Hz sweep width, 1.00 s acquisition time, 1 s recycle delay and using a 90 degree excitation pulse of approximately 11.5 μs at 100 W. Samples were analysed in MeOD-d_4 and chemical shifts reported in ppm (δ) relative to the solvent residual for methanol at 49.00 ppm.

Infrared spectra were recorded on a Nicolet 6700 FT-IR spectrometer using a diamond Smart iTR ATR attachment. Absorption maxima (λ_{max}) are described as s (strong), m (medium), w (weak), or br (broad) and are quoted in wavenumbers (cm^{-1}).

Accurate mass spectra for amphiphilic compounds were recorded on a Bruker AUtoflex III MALDI–TOF/TOF mass spectrometer. Experiment employed: positive ion, reflectron mode. The matrix employed was HCCA (α-cyano-4-hydroxycinnamic acid).

Copper analysis was carried out on an Agilent 770 ICP-MS. Nitric acid at ca. 100 °C was used to digest the samples before being made up to 25 mL with Milli Q water. The solutions were then measured directly by ICP-MS.

Synthesis of double-chain tails

Prop-2-yn-1-yl 3-hydroxy-2-(hydroxymethyl)-2-methylpropanoate (2)[1]

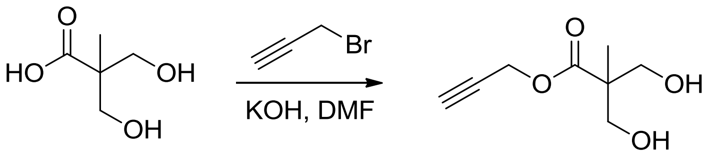

To a stirred solution of 2,2-bis(hydroxymethyl)propionic acid (1.0 equiv) in DMF (5 mL/mmol) was added KOH (1.1 equiv). After stirring for 2 h at 100 °C, propargyl bromide (1.0 equiv) was added dropwise over 30 min. The solution was heated at 80 °C for 18 h, before the reaction mixture was cooled, filtered and concentrated *in vacuo*. The crude product was diluted with ether and subsequently washed with brine and sat. aq. $NaHCO_3$ solution. The organic fragment was dried over $MgSO_4$ and concentrated *in vacuo* to give the title compound (4.06 g, 69%) without the need for further purification. Spectroscopic data matched with the literature data.

General procedure A

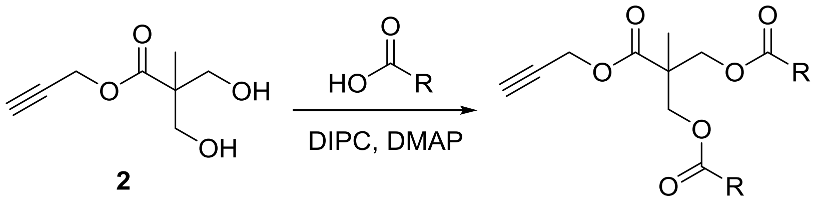

2

3-13

To a stirred solution of carboxylic acid (2.5 equiv) and DMAP (0.2 equiv) in DCM at r.t. (5 mL/mmol) was added diisopropylcarbodiimide (2.2 equiv). Once dissolved, diol **2** (1.0 equiv) was added and stirring continued for 18 h. The reaction mixture was then diluted with DCM and washed with 1.0 M aq. HCl, sat. aq. $NaHCO_3$ solution and brine, dried over $MgSO_4$ and concentrated *in vacuo* to give the crude material.

2-Methyl-2-((prop-2-yn-1-yloxy)carbonyl)propane-1,3-diyl dioctanoate (3)

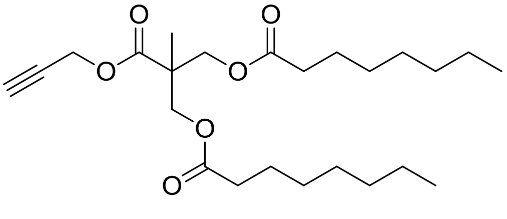

Synthesised in accordance with General Procedure A. Purification by column chromatography (4:1, petrol:ethyl acetate) afforded the compound (446 mg, 72%) as a yellow, viscous liquid; ν_{max}/cm^{-1} (thin film) 2927m, 2856m, 1738s, 1467m, 1378s, 1225m; δ_H (400 MHz, $CDCl_3$) 0.86 (6H, t, *J* 7.1, 2× CH_2-C**H₃**), 1.20-1.32 (19H, m, C**H₃** and 8×C**H₂**) 1.52-1.63 (4H, m, 2×O=C-CH_2-C**H₂**), 2.28 (4H, t, *J* 7.8, 2×O=C-C**H₂**), 2.43 (1H, t, *J* 2.2, ≡C**H**) 4.21 (2H, s, C-C**H₂**-O-), 4.22 (2H, s, C-C**H₂**-O-), 4.69 (2H, d, *J* 2.2, ≡C-C**H₂**-O-); δ_C (100 MHz, $CDCl_3$) 14.2 (2×CH_2-**C**H_3), 17.8 (C-**C**H_3), 22.7 (2×**C**H_2-CH_3), 25.0 (2×**C**H_2), 29.0 (2×**C**H_2), 29.2 (2×**C**H_2), 31.8 (2×**C**H_2), 34.3 (2×**C**H_2), 46.5 (**C**R_4), 52.7 (≡C-**C**H_2-O-), 65.3 (2×C-**C**H_2-O-), 75.3 (≡**C**H), 77.4 (HC≡**C**-), 172.3 (O=**C**), 173.4 (2×O=**C**); Accurate mass (ESI): Found: 425.2897 $C_{24}H_{41}O_6$ (MH^+) requires 425.2903.

2-Methyl-2-((prop-2-yn-1-yloxy)carbonyl)propane-1,3-diyl bis(decanoate) (4)

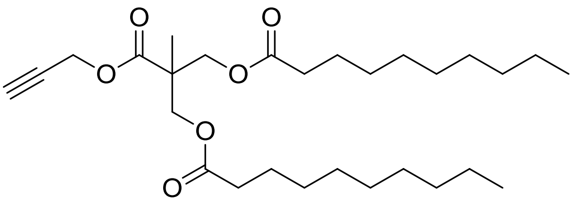

Synthesised in accordance with General Procedure A. Purification by column chromatography (5:1, petrol/ethyl acetate) afforded the compound (494 mg, 71%) as a colourless, viscous liquid; ν_{max}/cm^{-1} (thin film) 2924m, 2854m, 1740s, 1466m, 1377s, 1235m; δ_H (400 MHz, $CDCl_3$) 0.86 (6H, t, *J* 6.1, 2× CH_2-C**H₃**), 1.25 (27H, br s, C**H₃** and 12×C**H₂**), 1.53-1.63 (4H, m, 2× O=C-CH_2-C**H₂**), 2.28 (4H, t, *J* 7.8, 2× O=C-C**H₂**), 2.41-2.45 (1H, m, ≡C**H**) 4.21 (2H, s, C-C**H₂**-O-), 4.22 (2H, s, C-C**H₂**-O-), 4.69 (2H, d, *J* 3.0, ≡C-C**H₂**-O-); δ_C (100 MHz, $CDCl_3$) 14.3 (2×CH_2-**C**H_3), 17.8 (C-**C**H_3), 22.8 (2×**C**H_2-CH_3), 25.0 (2×**C**H_2), 29.3 (2×**C**H_2), 29.4 (2×**C**H_2), 29.6 (2×**C**H_2), 32.0 (2×**C**H_2), 34.3 (2×**C**H_2), 46.5 (**C**R_4), 52.7 (≡C-**C**H_2-O-), 65.3 (2×C-**C**H_2-O-), 75.3 (≡**C**H), 77.3 (HC≡**C**-), 172.3 (O=**C**), 173.4 (2×O=**C**); Accurate mass (ESI): Found: 481.3524 $C_{28}H_{49}O_6$ (MH^+) requires 481.3524.

2-Methyl-2-((prop-2-yn-1-yloxy)carbonyl)propane-1,3-diyl bis(dodecanoate) (5)

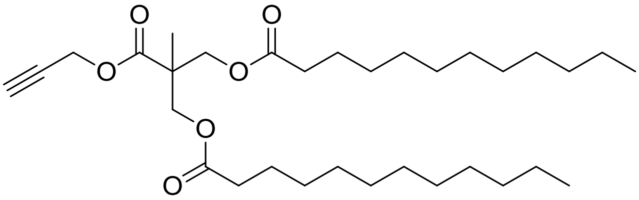

Synthesised in accordance with General Procedure A. Purification by column chromatography (5:1, petrol:ether) afforded the compound (373 mg, 70%) as a colourless, viscous liquid; ν_{max}/cm^{-1} (thin film) 2922m, 2853m, 1740s, 1466m, 1376s, 1232m; δ_H (400 MHz, $CDCl_3$) 0.85 (6H, t, *J* 6.7, 2× CH_2-C**H₃**), 1.19-1.33 (35H, m, C**H₃** & 16×C**H₂**) 1.52-1.62 (4H, m, 2×O=C-CH_2-C**H₂**), 2.27 (4H, t, *J* 7.5, 2×O=C-C**H₂**), 2.41-2.45 (1H, m, ≡C**H**) 4.21 (2H, s, C-C**H₂**-O-), 4.22 (2H, s, C-C**H₂**-O-), 4.68 (2H, d, *J* 2.5, ≡C-C**H₂**-O-); δ_C (100 MHz, $CDCl_3$) 14.3 (2×CH_2-**C**H_3), 17.8 (C-**C**H_3), 22.8 (2×**C**H_2-CH_3), 25.0 (2×**C**H_2), 29.3 (2×**C**H_2), 29.4 (2×**C**H_2), 29.5 (2×**C**H_2), 29.6 (2×**C**H_2), 29.8 (2×**C**H_2), 32.1 (2×**C**H_2), 34.3 (2×**C**H_2), 46.5 (**C**R_4), 52.7 (≡C-**C**H_2-O-), 65.3 (2×C-**C**H_2-O-), 75.3 (≡**C**H), 77.3 (HC≡**C**-), 172.3 (O=**C**), 173.4 (2×O=**C**); Accurate mass (ESI): Found: 537.4153 $C_{32}H_{57}O_6$ (MH^+) requires 537.4150.

2-Methyl-2-((prop-2-yn-1-yloxy)carbonyl)propane-1,3-diyl bis(tetradecanoate) (6)

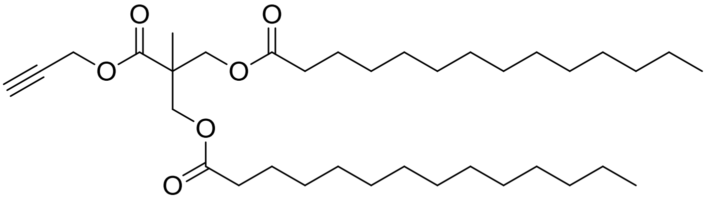

Synthesised in accordance with General Procedure A. Purification by column chromatography (5:1, petrol/ether) afforded the compound (337 mg, 56%) as a colourless, viscous liquid; ν_{max}/cm^{-1} (thin film) 2921m, 2851m, 1740s, 1467m, 1377s, 1237m; δ_H (400 MHz, $CDCl_3$) 0.85 (6H, t, *J* 6.7, 2× CH_2-C**H₃**), 1.18-1.33 (43H, m, C**H₃** and 20×C**H₂**), 1.52-1.62 (4H, m, 2×O=C-CH_2-C**H₂**), 2.27 (4H, t, *J* 7.5, 2×O=C-C**H₂**), 2.43 (1H, t, *J* 2.8, ≡C**H**), 4.21 (2H, s, C-C**H₂**-O-), 4.22 (2H, s, C-C**H₂**-O-), 4.68 (2H, d, *J* 2.8, ≡C-C**H₂**-O-); δ_C (100 MHz, $CDCl_3$) 14.3 (2×CH_2-**C**H_3), 17.8 (C-**C**H_3), 22.8 (2×**C**H_2-CH_3), 25.0 (2×**C**H_2), 29.3 (2×**C**H_2), 29.4 (2×**C**H_2), 29.5 (2×**C**H_2), 29.6 (2×**C**H_2), 29.8 (2×**C**H_2), 29.8 (2×**C**H_2), 29.9 (2×**C**H_2), 32.1 (2×**C**H_2), 34.3 (2×**C**H_2), 46.5 (**C**R_4), 52.7 (≡C-**C**H_2-O-), 65.3 (2×C-

CH_2-O-), 75.3 (≡**C**H), 77.3 (HC≡**C**-), 172.3 (O=**C**), 173.4 (2×O=**C**); Accurate mass (ESI): Found: 593.4774 $C_{36}H_{65}O_6$ (MH^+) requires 593.4776.

2-Methyl-2-((prop-2-yn-1-yloxy)carbonyl)propane-1,3-diyl bis(hexadecanoate) (7)

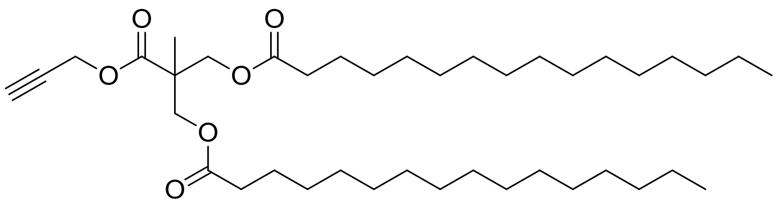

Synthesised in accordance with General Procedure A. Purification by column chromatography (5:1, petrol/ether) afforded the compound (410 mg, 62%) as a white solid; ν_{max}/cm^{-1} (thin film) 2916m, 2849m, 1732s, 1463m, 1378s, 1245m; δ_H (400 MHz, $CDCl_3$) 0.86 (6H, t, *J* 6.8, 2× CH_2-C**H₃**), 1.18-1.34 (51H, m, C**H₃** & 24×C**H₂**) 1.53-1.63 (4H, m, 2×O=C-CH_2-C**H₂**), 2.27 (4H, t, *J* 7.1, 2×O=C-C**H₂**), 2.43 (1H, t, *J* 2.4, ≡C**H**) 4.21 (2H, s, C-C**H₂**-O-), 4.22 (2H, s, C-C**H₂**-O-), 4.68 (2H, d, *J* 2.4, ≡C-C**H₂**-O-); δ_C (100 MHz, $CDCl_3$) 14.3 (2×CH_2-**C**H_3), 17.8 (C-**C**H_3), 22.9 (2×**C**H_2-CH_3), 25.0 (2×**C**H_2), 29.3 (2×**C**H_2), 29.4 (2×**C**H_2), 29.5 (2×**C**H_2), 29.6 (2×**C**H_2), 29.8 (2×**C**H_2), 29.8 (4×**C**H_2), 29.9 (4×**C**H_2), 32.1 (2×**C**H_2), 34.3 (2×**C**H_2), 46.5 (**C**R_4), 52.7 (≡C-**C**H_2-O-), 65.3 (2×C-**C**H_2-O-), 75.3 (≡**C**H), 77.3 (HC≡**C**-), 172.3 (O=**C**), 173.4 (2×O=**C**); Accurate mass (ESI): Found: 649.5399 $C_{40}H_{73}O_6$ (MH^+) requires 649.5402.

2-Methyl-2-((prop-2-yn-1-yloxy)carbonyl)propane-1,3-diyl bis(octadecanoate) (8)

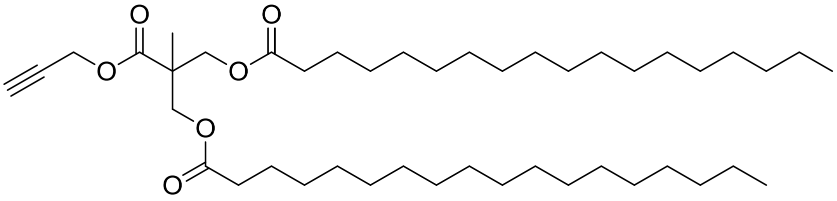

Synthesised in accordance with General Procedure A. Purification by column chromatography (5:1, petrol/ether) afforded the compound (410 mg, 62%) as a white solid; ν_{max}/cm^{-1} (thin film) 2915m, 2849m, 1738s, 1467m, 1380s, 1235m; δ_H (400 MHz, $CDCl_3$) 0.86 (6H, t, *J* 6.8, 2× CH_2-C**H₃**), 1.18-1.33 (59H, m, C**H₃** and 28×C**H₂**) 1.53-1.61 (4H, m, 2×O=C-CH_2-C**H₂**), 2.27 (4H, t, *J* 7.4, 2×O=C-

C**H$_2$**), 2.43 (1H, t, *J* 2.4, ≡C**H**) 4.21 (2H, s, C-C**H$_2$**-O-), 4.22 (2H, s, C-C**H$_2$**-O-), 4.68 (2H, d, *J* 2.4, ≡C-C**H$_2$**-O-); δ_C (100 MHz, $CDCl_3$) 14.3 (2×CH_2-**C**H_3), 17.8 (C-**C**H_3), 22.9 (2×**C**H_2-CH_3), 25.0 (2×**C**H_2), 29.3 (2×**C**H_2), 29.4 (2×**C**H_2), 29.8 (2×**C**H_2), 29.6 (2×**C**H_2), 29.8 (2×**C**H_2), 29.8 (4×**C**H_2), 29.9 (8×**C**H_2), 32.1 (2×**C**H_2), 34.3 (2×**C**H_2), 46.8 (**C**R_4), 52.7 (≡C-**C**H_2-O-), 65.3 (2×C-**C**H_2-O-), 75.3 (≡**C**H), 77.3 (HC≡**C**-), 172.3 (O=**C**), 173.4 (2×O=**C**).

2-Methyl-2-((prop-2-yn-1-yloxy)carbonyl)propane-1,3-diyl bis(3,7,11,15-tetramethylhexadecanoate) (9)

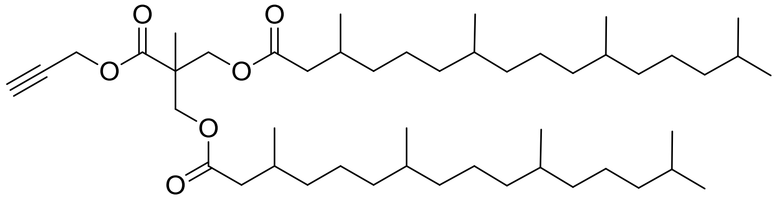

Synthesised in accordance with General Procedure A. Purification by column chromatography (5:1, petrol/ether) afforded the compound (303 mg, 52%) as a colourless, viscous oil; ν_{max}/cm^{-1} (thin film) 2953m, 2925m, 2868m, 1741s, 1462m, 1377m, 1236m; δ_H (400 MHz, $CDCl_3$) 0.79-0.87 (24H, app. dd, *J* 6.8, 6.5, 8×CH-C**H$_3$**), 0.89 (6H, d, *J* 6.5, 2×O=C-CH_2-CH-C**H$_3$**), 0.99-1.40 (43H, m, C**H$_3$** and 18×C**H$_2$** and 4×C**H**), 1.50 (2H, m, *J* 6.8, 2×CH_2-C**H**-$(CH_3)_2$), 1.83-1.96 (2H, m, 2×O=C-CH_2-C**H**), 2.08 (2H, dd, *J* 14.5, 8.3, 2×O=C-C**H**H'-CH), 2.29 (2H, dd, *J* 15.1, 5.8, 2×O=C-CH**H'**-CH), 2.43 (1H, t, *J* 2.5, ≡C**H**), 4.19-4.24 (4H, s, 2×C-C**H$_2$**-O-), 4.68 (2H, d, *J* 2.5, ≡C-C**H$_2$**-O-); δ_C (100 MHz, $CDCl_3$) 17.8 (C-**C**H_3), 19.8 (CH-**C**H_3), 19.8 (CH-**C**H_3), 19.8 (2×CH-**C**H_3), 19.9 (CH-**C**H_3), 19.9 (CH-**C**H_3), 22.8 (2×CH-**C**H_3), 22.9 (2×CH-**C**H_3), 24.5 (2×CH_2-**C**H_2-CH_2), 24.6 (2×CH_2-**C**H_2-CH_2), 25.0 (2×CH_2-**C**H_2-CH_2), 28.1 (3×R_3**C**H), 30.5 (2×R_3**C**H), 32.9 (3×R_3**C**H), 37.2 (2×**C**H_2-CH), 37.3 (**C**H_2-CH), 37.4 (**C**H_2-CH), 37.4 (2×**C**H_2-CH), 37.6 (2×**C**H_2-CH), 37.6 (2×**C**H_2-CH), 39.5 (2×**C**H_2-CH-$(CH_3)_2$), 41.8 (O=C-**C**H_2-CH), 41.8 (O=C-**C**H_2-CH), 46.4 (**C**R_4), 52.7 (≡C-**C**H_2-O-), 65.3 (2×C-**C**H_2-O-), 75.3 (≡**C**H), 77.3 (HC≡**C**-), 172.2 (O=**C**), 172.9 (2×O=**C**); Accurate mass (ESI): Found: 761.6643 $C_{48}H_{89}O_6$ (MH^+) requires 761.6661.

(7*Z*,7'*Z*)-2-Methyl-2-((prop-2-yn-1-yloxy)carbonyl)propane-1,3-diyl bis(hexadec-7-enoate) (10)

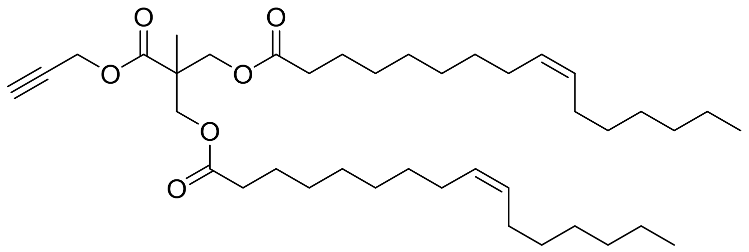

Synthesised in accordance with General Procedure A. Purification by column chromatography (5:1, petrol/ether) afforded the compound (410 mg, 62%) as a colourless, viscous oil; ν_{max}/cm^{-1} (thin film) 2916m, 2849m, 1733s, 1473m, 1463m, 1235m, 1213m; δ_H (400 MHz, CDCl$_3$) 0.86 (6H, t, *J* 6.5, 2× CH$_2$-C**H$_3$**), 1.20-1.34 (35H, m, C**H$_3$** & 16×C**H$_2$**) 1.52-1.62 (4H, m, 2×O=C-CH$_2$-C**H$_2$**), 1.94-2.04 (8H, m, 4×=CH-C**H$_2$**), 2.28 (4H, t, *J* 7.4, 2×O=C-C**H$_2$**), 2.43 (1H, t, *J* 2.2, ≡C**H**), 4.21 (2H, s, C-C**H$_2$**-O), 4.22 (2H, s, C-C**H$_2$**-O), 4.68 (2H, d, *J* 2.2, ≡C-C**H$_2$**-O), 5.32 (4H, dt, *J* 5.6, 3.6, 2×C**H**=C**H**); δ_C (100 MHz, CDCl$_3$) 14.3 (2×CH$_2$-**C**H$_3$), 17.8 (C-**C**H$_3$), 22.8 (2×**C**H$_2$-CH$_3$), 25.0 (2×**C**H$_2$), 27.3 (2×=C-**C**H$_2$), 27.4 (2×**C**H$_2$-C=), 29.1 (2×**C**H$_2$), 29.3 (2×**C**H$_2$), 29.3 (2×**C**H$_2$), 29.9 (2×**C**H$_2$), 29.9 (2×**C**H$_2$), 31.9 (2×**C**H$_2$), 34.3 (2×**C**H$_2$), 46.8 (**C**R$_4$), 52.7 (≡C-**C**H$_2$-O-), 65.3 (2×C-**C**H$_2$-O-), 75.3 (≡**C**H), 77.3 (HC≡**C**-), 129.9 (2×C=**C**), 130.1 (2×**C**=C), 172.2 (O=**C**), 173.4 (2×O=**C**); Accurate mass (ESI): Found: 667.4906 $C_{40}H_{68}O_6Na$ (MNa$^+$) requires 667.4908.

(*Z*)-2-Methyl-2-((prop-2-yn-1-yloxy)carbonyl)propane-1,3-diyl dioleate (11)

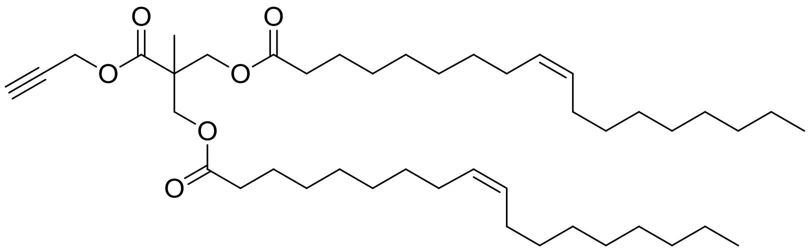

Synthesised in accordance with General Procedure A. Purification by column chromatography (5:1, petrol/ether) afforded the compound (464 mg, 76%) as a colourless, viscous oil; ν_{max}/cm^{-1} (thin film) 2922m, 2853m, 1742s, 1465m, 1463m, 1377m, 1237m; δ_H (400 MHz, CDCl$_3$) 0.86 (6H, t, *J* 6.8, 2× CH$_2$-C**H$_3$**), 1.19-1.34 (47H, m, C**H$_3$** and 22×C**H$_2$**) 1.53-1.62 (4H, m, 2×O=C-CH$_2$-C**H$_2$**), 1.95-2.02 (8H, m, 4×=CH-C**H$_2$**), 2.28 (4H, t, *J* 6.2, 2×O=C-C**H$_2$**), 2.43 (1H, t, *J* 2.5, ≡C**H**) 4.21 (2H, s, C-C**H$_2$**-

O-), 4.22 (2H, s, C-C**H₂**-O-), 4.68 (2H, d, *J* 2.5, ≡C-C**H₂**-O-), 5.32 (4H, dt, *J* 5.6, 3.4, 2×-C**H**=C**H-**); δ_C (100 MHz, $CDCl_3$) 14.3 (2×CH_2-**C**H_3), 17.8 (C-**C**H_3), 22.8 (2×**C**H_2-CH_3), 25.0 (2×**C**H_2), 27.3 (2×=C-**C**H_2), 27.4 (2×**C**H_2-C=), 29.3 (2×**C**H_2), 29.3 (2×**C**H_2), 29.3 (2×**C**H_2), 29.5 (2×**C**H_2), 29.7 (2×**C**H_2), 29.9 (2×**C**H_2), 29.9 (2×**C**H_2), 32.1 (2×**C**H_2), 34.3 (2×**C**H_2), 46.5 (**C**R_4), 52.7 (≡C-**C**H_2-O-), 65.3 (2×C-**C**H_2-O-), 75.3 (≡**C**H), 77.3 (HC≡**C**-), 129.9 (2×C=**C**) 130.2 (2×**C**=C), 172.2 (O=**C**), 173.4 (2×O=**C**); Accurate mass (ESI): Found: 701.5712 $C_{44}H_{77}O_6$ (MH^+) requires 701.5715.

(*Z*)-2-Methyl-2-((prop-2-yn-1-yloxy)carbonyl)propane-1,3-diyl bis(docos-13-enoate) (12)

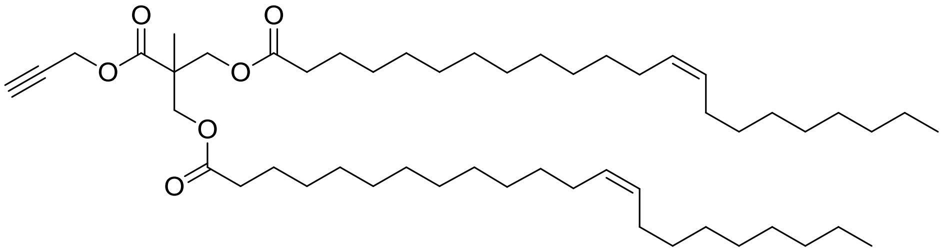

Synthesised in accordance with General Procedure A. Purification by column chromatography (5:1, petrol/ether) afforded the compound (487 mg, 69%) as a colourless, viscous oil; ν_{max}/cm^{-1} (thin film) 2921m, 2852m, 1743s, 1465m, 1377m, 1236m; δ_H (400 MHz, $CDCl_3$) 0.86 (6H, t, *J* 6.5, 2×CH_2-C**H₃**), 1.19-1.34 (63H, m, C**H₃** and 30×C**H₂**) 1.52-1.61 (4H, m, 2×O=C-CH_2-C**H₂**), 1.99 (8H, q, *J* 6.4, 4×=CH-C**H₂**), 2.28 (4H, t, *J* 7.3, 2×O=C-C**H₂**), 2.43 (1H, t, *J* 2.5, ≡C**H**), 4.21 (2H, s, C-C**H₂**-O-), 4.22 (2H, s, C-C**H₂**-O-), 4.69 (2H, d, *J* 2.5, ≡C-C**H₂**-O-), 5.32 (4H, *J* 8.9, 4.5, 2×-C**H**=C**H-**); δ_C (100 MHz, $CDCl_3$) 14.3 (2×CH_2-**C**H_3), 17.8 (C-**C**H_3), 22.8 (2×**C**H_2-CH_3), 25.0 (2×**C**H_2), 27.4 (4×=C-**C**H_2), 29.3 (2×**C**H_2), 29.4 (2×**C**H_2), 29.5 (2×**C**H_2), 29.6 (2×**C**H_2), 29.7 (4×**C**H_2), 29.7 (2×**C**H_2), 29.8 (2×**C**H_2), 29.9 (2×**C**H_2), 32.1 (2×**C**H_2), 34.3 (2×**C**H_2), 46.5 (**C**R_4), 52.7 (≡C-**C**H_2-O-), 65.3 (2×C-**C**H_2-O-), 75.3 (≡**C**H), 77.3 (HC≡**C**-), 130.0 (2×C=**C**) 130.1 (2×**C**=C), 172.3 (O=**C**), 173.4 (2×O=**C**); Accurate mass (ESI): Found: 813.6971 $C_{52}H_{93}O_6$ (MH^+) requires 813.6974.

(9*Z*,9'*Z*,12*Z*,12'*Z*)-2-Methyl-2-((prop-2-yn-1-yloxy)carbonyl)propane-1,3-diyl bis(octadeca-9,12-dienoate) (13)

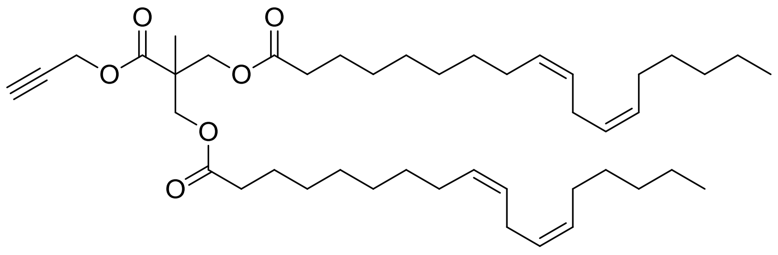

Synthesised in accordance with General Procedure A. Purification by column chromatography (5:1, petrol/ether) afforded the compound (192 mg, 40%) as a colourless, viscous oil; ν_{max}/cm^{-1} (thin film) 2925m, 2852m, 1742s, 1465m, 1377m, 1236m; δ_H (400 MHz, $CDCl_3$) 0.86 (6H, t, *J* 6.7, 2×CH_2-C$\mathbf{H_3}$), 1.20-1.38 (35H, m, C$\mathbf{H_3}$ and 16×C$\mathbf{H_2}$) 1.53-1.62 (4H, m, 2×O=C-CH_2-C$\mathbf{H_2}$), 2.02 (8H, q, *J* 6.7, 4×=CH-C$\mathbf{H_2}$), 2.28 (4H, t, *J* 7.4, 2×O=C-C$\mathbf{H_2}$), 2.43 (1H, t, *J* 2.5, ≡C**H**), 2.75 (4H, t, *J* 6.5, C=C-C$\mathbf{H_2}$-C=C), 4.21 (2H, s, C-C$\mathbf{H_2}$-O-), 4.22 (2H, s, C-C$\mathbf{H_2}$-O-), 4.69 (2H, d, *J* 2.5, ≡C-C$\mathbf{H_2}$-O-), 5.26-5.40 (8H, ,m, 4×-C**H**=C**H-**); δ_C (100 MHz, $CDCl_3$) 14.2 (2×CH_2-**C**H_3), 17.8 (C-**C**H_3), 22.7 (2×**C**H_2-CH_3), 25.0 (2×**C**H_2), 25.8 (2×=C-**C**H_2-C=), 27.4 (4×=C-**C**H_2), 29.2 (2×**C**H_2), 29.3 (2×**C**H_2), 29.3 (2×**C**H_2), 29.5 (2×**C**H_2), 29.8 (2×**C**H_2), 31.7 (2×**C**H_2), 34.2 (2×**C**H_2), 46.5 (**C**R_4), 52.7 (≡C-**C**H_2-O-), 65.3 (2×C-**C**H_2-O-), 75.3 (≡**C**H), 77.3 (HC≡**C**-), 128.1 (2×C=**C**) 128.2 (2×**C**=C), 130.2 (2×C=**C**), 130.4 (2×**C**=C), 172.2 (O=**C**), 173.4 (2×O=**C**); Accurate mass (ESI): Found: 697.5385 $C_{44}H_{73}O_6$ (MH^+) requires 697.5405.

Synthesis of triple chain tails

N-(1,3-Dihydroxy-2-(hydroxymethyl)propan-2-yl)pent-4-ynamide (15)

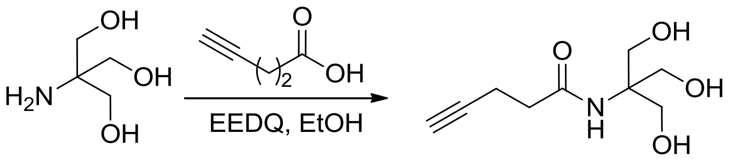

To a stirred solution of tri-(hydroxymethyl)-methylamine (TRIS) (1.1 equiv) and *N*-ethoxycarbonyl-2-ethoxy-1,2-dihydroquinoline (EEDQ) (1.2 equiv) in ethanol (5 mL/mmol) was added 4-pentynoic acid (1.0 equiv). The reaction mixture was heated to 60 °C and stirred for 20 h. The reaction mixture was cooled, filtered and concentrated in vacuo to give the crude material. Purification by column chromatography (5:2 ethyl acetate/petrol) afforded the compound (951 mg, 84%) as a white solid; ν_{max}/cm^{-1} (thin film) 3446br, 2935br, 1656s, 1389m, 1215m; δ_H (400 MHz, MeOD) 2.25 (1H, s, ≡C**H**), 2.44 (4H, m, ≡C-C**H₂**-C**H₂**-C=O), 3.70 (6H, s, 3×C**H₂**-OH); δ_C (100 MHz, $CDCl_3$) 14.4 (≡C-**C**H_2), 35.2 (**C**H_2- C=O), 61.3 (3×**C**H_2-OH), 62.5 (**C**-NH), 69.1 (≡**C**H), 82.4 (≡**C**-CH_2), 173.8 (O=**C**); Accurate mass (ESI): Found: 202.1076 $C_9H_{16}O_4N$ (MH^+) requires 202.1079.

General procedure B

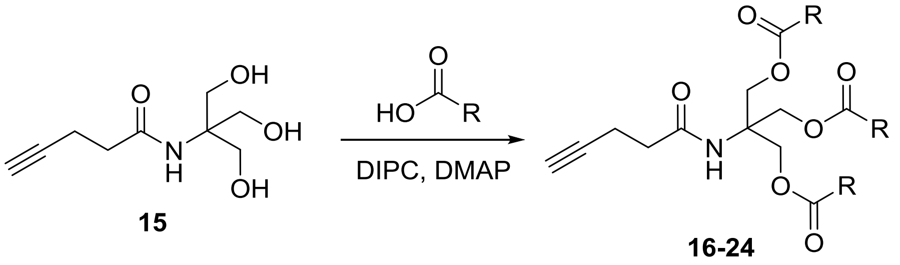

To a stirred solution of carboxylic acid (3.5 equiv) and DMAP (0.3 equiv) in DCM at rt (5 mL/mmol) was added diisopropylcarbodiimide (3.3 equiv). Once dissolved, propargyl amide **15** (1.0 equiv) was added and stirring continued for 18 h. The reaction was then diluted with DCM and washed with 1.0 M aq. HCl, sat. aq. $NaHCO_3$ solution and brine, dried over $MgSO_4$ and concentrated in vacuo to give the crude product.

2-((Octanoyloxy)methyl)-2-(pent-4-ynamido)propane-1,3-diyl dioctanoate (16)

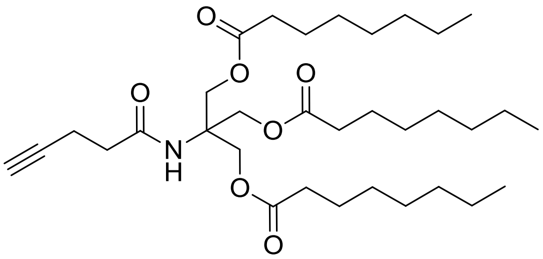

Synthesised in accordance with General Procedure B. Purification by column chromatography (5:1, petrol/ethyl acetate) afforded the compound (556 mg, 77%) as a colourless, viscous liquid; ν_{max}/cm^{-1} (thin film) 3313br, 2924m, 2855m, 1737s, 1661s, 1546m, 1464m, 1379m; δ_H (400 MHz, CDCl$_3$) 0.86 (9H, t, *J* 6.1, 3× CH$_2$-C**H$_3$**), 1.19-1.34 (24H, m, 12×C**H$_2$**) 1.53-1.65 (6H, m, 3×O=C-CH$_2$-C**H$_2$**), 1.95 (1H, t, *J* 2.5, ≡C**H**), 2.26-2.37 (8H, m, ≡C-CH$_2$-C**H$_2$**, overlays t, *J* 7.8, 3×O=C-C**H$_2$**), 2.41-2.47 (2H, m, ≡C-C**H$_2$**), 4.40 (6H, s, 3×C**H$_2$**-O-) 6.02 (1H, br s, N**H**); δ_C (100 MHz, CDCl$_3$) 14.2 (3×CH$_2$-**C**H$_3$), 14.9 (≡C-CH$_2$-**C**H$_2$), 22.7 (3×**C**H$_2$-CH$_3$), 25.0 (3×**C**H$_2$), 29.1 (3×**C**H$_2$), 29.2 (3×**C**H$_2$), 31.8 (3×**C**H$_2$), 34.2 (3×**C**H$_2$), 36.0 (≡C-**C**H$_2$-CH$_2$), 58.6 (**C**R$_3$N), 62.7 (3×C-**C**H$_2$-O-), 69.5 (≡**C**H), 82.8 (HC≡**C**-), 171.2 (O=**C**), 173.6 (3×O=**C**); Accurate mass (ESI): Found: 580.4212 $C_{33}H_{58}O_7N$ (MH$^+$) requires 580.4213.

2-((Decanoyloxy)methyl)-2-(pent-4-ynamido)propane-1,3-diyl bis(decanoate) (17)

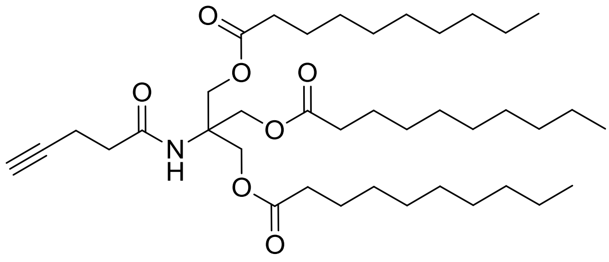

Synthesised in accordance with General Procedure B. Purification by column chromatography (6:1, petrol/ethyl acetate) afforded the compound (520 mg, 72%) as a white solid; ν_{max}/cm^{-1} (thin film) 2923m, 2854m, 1740s, 1686s, 1544m, 1466m, 1378m, 1241m; δ_H (400 MHz, CDCl$_3$) 0.85 (9H, t, *J* 6.8, 3× CH$_2$-C**H$_3$**), 1.19-1.33 (36H, m, 18×C**H$_2$**) 1.53-1.63 (6H, m, 3×O=C-CH$_2$-C**H$_2$**), 1.96 (1H, t, *J* 2.8, ≡C**H**), 2.26-2.37 (8H, m, ≡C-CH$_2$-C**H$_2$,** overlays t, *J* 7.1, 3×O=C-C**H$_2$**), 2.43-2.48 (2H, m, ≡C-

C**H₂**), 4.41 (6H, s, 3×C**H₂**-O-), 6.02 (1H, br s, N**H**); δ_C (100 MHz, $CDCl_3$) 14.3 (3×CH_2-**C**H_3), 14.9 (≡C-CH_2-**C**H_2), 22.8 (3×**C**H_2-CH_3), 25.0 (3×**C**H_2), 29.3 (3×**C**H_2), 29.4 (6×**C**H_2), 29.6 (3×**C**H_2), 32.0 (3×**C**H_2), 34.3 (3×**C**H_2), 36.0 (≡C-**C**H_2-CH_2), 58.7 (**C**R_3N), 62.7 (3×C-**C**H_2-O-), 69.5 (≡**C**H), 82.8 (HC≡**C**-), 171.2 (O=**C**), 173.6 (3×O=**C**); Accurate mass (ESI): Found: 664.5148 $C_{39}H_{70}O_7N$ (MH^+) requires 664.5147.

2-((Dodecanoyloxy)methyl)-2-(pent-4-ynamido)propane-1,3-diyl didodecanoate (18)

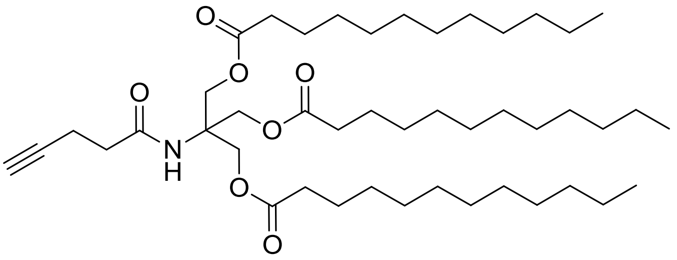

Synthesised in accordance with General Procedure B. Purification by column chromatography (6:1, petrol/ethyl acetate) afforded the compound (527 mg, 71%) as a white solid; ν_{max}/cm^{-1} (thin film) 2917s, 2849s, 1735s, 1651s, 1557m, 1468m, 1373m, 1269m; δ_H (400 MHz, $CDCl_3$) 0.86 (9H, t, *J* 6.7, 3× CH_2-C**H₃**), 1.20-1.33 (48H, m, 24×C**H₂**) 1.54-1.63 (6H, m, 3×O=C-CH_2-C**H₂**), 1.96 (1H, t, *J* 2.5, ≡C**H**), 2.27-2.37 (8H, m, ≡C-CH_2-C**H₂**, overlays t, *J* 7.4, 3×O=C-C**H₂**), 2.43-2.49 (2H, m, ≡C-C**H₂**), 4.41 (6H, s, 3×C**H₂**-O-), 6.03 (1H, br s, N**H**); δ_C (100 MHz, $CDCl_3$) 14.3 (3×CH_2-**C**H_3), 14.9 (≡C-CH_2-**C**H_2), 22.8 (3×**C**H_2-CH_3), 25.0 (3×**C**H_2), 29.3 (3×**C**H_2), 29.4 (3×**C**H_2), 29.5 (3×**C**H_2), 29.6 (3×**C**H_2), 29.8 (3×**C**H_2) 32.1 (3×**C**H_2), 34.3 (3×**C**H_2), 36.0 (≡C-**C**H_2-CH_2), 58.7 (**C**R_3N), 62.7 (3×C-**C**H_2-O-), 69.5 (≡**C**H), 82.8 (HC≡**C**-), 171.2 (O=**C**), 173.6 (3×O=**C**); Accurate mass (ESI): Found: 748.6082 $C_{45}H_{82}O_7N$ (MH^+) requires 748.6086.

2-(Pent-4-ynamido)-2-((tetradecanoyloxy)methyl)propane-1,3-diyl ditetradecanoate (19)

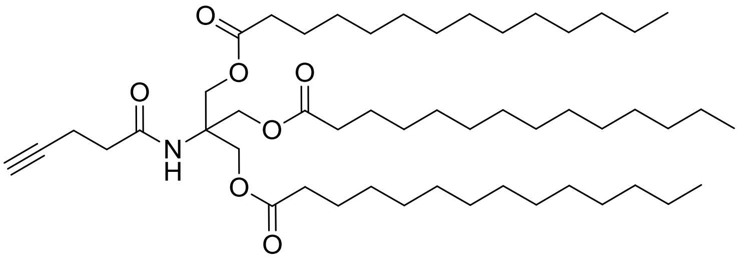

Synthesised in accordance with General Procedure B. Purification by column chromatography (3:1, petrol/ether) afforded the compound (492 mg, 79%) as a white solid; ν_{max}/cm^{-1} (thin film) 2916s, 2849s, 1736s, 1652s, 1555m, 1469m, 1373m, 1269m; δ_H (400 MHz, $CDCl_3$) 0.86 (9H, t, *J* 6.4, 3× CH_2-C**H₃**), 1.17-1.33 (60H, m, 30×C**H₂**) 1.54-1.63 (6H, m, 3×O=C-CH_2-C**H₂**), 1.96 (1H, t, *J* 2.7, ≡C**H**), 2.26-2.37 (8H, m, ≡C-CH_2-C**H₂,** overlays t, *J* 7.7, 3×O=C-C**H₂**), 2.43-2.49 (2H, m, ≡C-C**H₂**), 4.41 (6H, s, 3×C**H₂**-O-), 6.03 (1H, br s, N**H**); δ_C (100 MHz, $CDCl_3$) 14.3 (3×CH_2-**C**H_3), 14.9 (≡C-CH_2-**C**H_2), 22.9 (3×**C**H_2-CH_3), 25.0 (3×**C**H_2), 29.3 (3×**C**H_2), 29.4 (3×**C**H_2), 29.5 (3×**C**H_2), 29.6 (3×**C**H_2), 29.8 (6×**C**H_2), 29.8 (3×**C**H_2), 29.8 (3×**C**H_2), 32.1 (3×**C**H_2), 34.3 (3×**C**H_2), 36.0 (≡C-**C**H_2-CH_2), 58.7 (**C**R_3N), 62.7 (3×C-**C**H_2-O-), 69.5 (≡**C**H), 82.8 (HC≡**C**-), 171.2 (O=**C**), 173.6 (3×O=**C**); Accurate mass (ESI): Found: 832.7024 $C_{51}H_{94}O_7N$ (MH^+) requires 832.7025.

2-((Palmitoyloxy)methyl)-2-(pent-4-ynamido)propane-1,3-diyl dipalmitate (20)

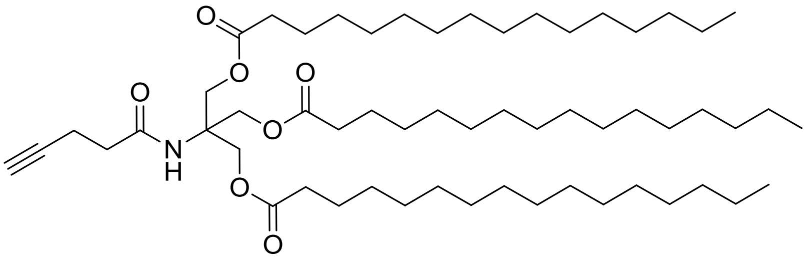

Synthesised in accordance with General Procedure B. Purification by column chromatography (5:1, petrol/ether) afforded the compound (442 mg, 61%) as a white solid; ν_{max}/cm^{-1} (thin film) 2916s, 2849s, 1736s, 1652s, 1552m, 1469m, 1373m, 1266m; δ_H (400 MHz, $CDCl_3$) 0.86 (9H, t, *J* 6.2, 3× CH_2-C**H₃**), 1.19-1.34 (72H, m, 36×C**H₂**) 1.54-1.63 (6H, m, 3×O=C-CH_2-C**H₂**), 1.96 (1H, t, *J* 2.7, ≡C**H**), 2.27-2.37 (8H, m, ≡C-CH_2-C**H₂**, overlays t, *J* 7.4, 3×O=C-C**H₂**), 2.43-2.50 (2H, m, ≡C-C**H₂**), 4.41 (6H, s, 3×C**H₂**-O-), 6.03 (1H, br s, N**H**); δ_C (100 MHz, $CDCl_3$) 14.3 (3×CH_2-**C**H_3), 14.9 (≡C-CH_2-**C**H_2), 22.9 (3×**C**H_2-CH_3), 25.0 (3×**C**H_2), 29.3 (3×**C**H_2), 29.4 (3×**C**H_2), 29.5 (3×**C**H_2), 29.7 (3×**C**H_2), 29.8 (6×**C**H_2), 29.8 (6×**C**H_2), 29.9 (6×**C**H_2), 32.1 (3×**C**H_2), 34.3 (3×**C**H_2), 36.0 (≡C-**C**H_2-CH_2), 58.7 (**C**R_3N), 62.7 (3×C-**C**H_2-O-), 69.5 (≡**C**H), 82.8 (HC≡**C**-), 171.2 (O=**C**), 173.6 (3×O=**C**); Accurate mass (ESI): Found: 916.7965 $C_{40}H_{73}O_6$ (MH^+) requires 916.7964.

2-(Pent-4-ynamido)-2-(((3,7,11,15-tetramethylhexadecanoyl)oxy)methyl)propane-1,3-diyl bis(3,7,11,15-tetramethylhexadecanoate) (21)

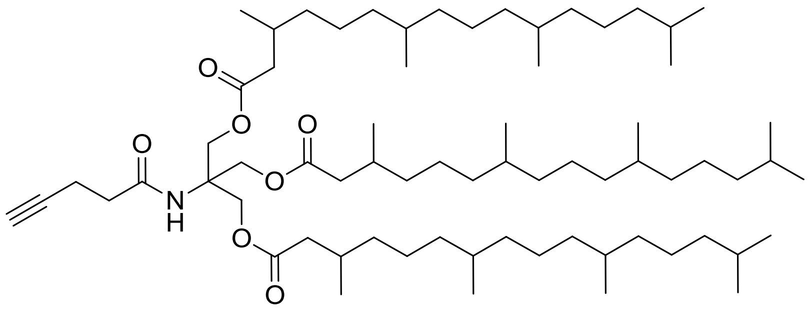

Synthesised in accordance with General Procedure B. Purification by column chromatography (5:1, petrol/ether) afforded the compound (293 mg, 61%) as a colourless, viscous oil; ν_{max}/cm^{-1} (thin film) 2953s, 2924s, 2868s, 1742s, 1658s, 1546m, 1462m, 1378m, 1244m; δ_H (400 MHz, $CDCl_3$) 0.81-0.87 (36H, app. dd, *J* 6.8, 6.5, 12×CH-C**H₃**), 0.91 (6H, d, *J* 6.5, 3×O=C-CH_2-CH-C**H₃**), 0.98-1.41 (60H, m, 27×C**H₂** and 6×C**H**), 1.50 (3H, app. non, *J* 6.5, 3×CH_2-C**H**-$(CH_3)_2$), 1.86-1.98 (3H, m, 3×O=C-CH_2-C**H** overlays t, *J* 2.7, ≡C**H**), 2.10 (3H, dd, *J* 14.4, 8.5, 3×O=C-C**H**H'-CH), 2.28-2.37 (9H, m, 3×O=C-CH**H'**-CH and 3×O=C-C**H₂**), 2.42-2.49 (2H, m, ≡C-C**H₂**), 4.42 (6H, s, 3×C**H₂**-O-), 6.06 (1H, br s, N**H**); δ_C (100 MHz, $CDCl_3$) 14.9 (≡C-CH_2-**C**H_2), 19.7 (**C**H_3), 19.8 (**C**H_3), 19.8 (2×**C**H_3), 19.8 (3×**C**H_3), 19.9 (**C**H_3), 19.9 (**C**H_3), 22.8 (3×**C**H_3) 22.9 (3×**C**H_3), 24.5 (3×**C**H_2), 24.6 (3×**C**H_2), 25.0 (3×**C**H_2), 28.1 (3×**C**H), 30.5 (3×**C**H), 32.9 (3×**C**H), 33.0 (3×**C**H), 36.0 (≡C-**C**H_2-CH_2), 37.2 (3×**C**H_2), 37.3 (**C**H_2), 37.4 (**C**H_2), 37.4 (2×**C**H_2), 37.5 (**C**H_2), 37.6 (2×**C**H_2), 37.6 (2×**C**H_2), 37.6 (2×**C**H_2), 37.7 (**C**H_2), 39.5 (3×**C**H_2-CH-$(CH_3)_2$), 41.7 (O=C-**C**H_2-CH), 41.8 (O=C-**C**H_2-CH), 41.8 (O=C-**C**H_2-CH), 58.7 (**C**R_3N), 62.7 (3×C-**C**H_2-O-), 69.5 (≡**C**H), 82.8 (HC≡**C**-), 171.1 (O=**C**), 173.1 (3×O=**C**); Accurate mass (ESI): Found: 1084.9813 $C_{69}H_{130}O_7N$ (MH^+) requires 1084.9849.

(7*Z*,7'*Z*)-2-(((*Z*)-hexadec-7-enoyloxy)methyl)-2-(pent-4-ynamido)propane-1,3-diyl bis(hexadec-7-enoate) (22)

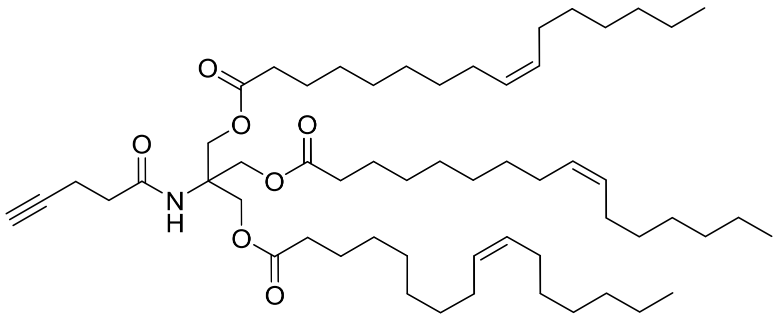

Synthesised in accordance with General Procedure B. Purification by column chromatography (5:1, petrol/ether) afforded the compound (240 mg, 53%) as a colourless, viscous oil; ν_{max}/cm^{-1} (thin film) 2923s, 2853s, 1743s, 1658s, 1545m, 1465m, 1378m, 1239m; δ_H (400 MHz, $CDCl_3$) 0.86 (9H, t, *J* 6.2, 3×CH_2-C**H**$_3$), 1.20-1.35 (48H, m, 24×C**H**$_2$) 1.54-1.64 (6H, m, 3×O=C-CH_2-C**H**$_2$), 1.94-2.04 (13H, m, 6×=CH-C**H**$_2$ overlays ≡C**H**), 2.26-2.38 (8H, m, ≡C-CH_2-C**H**$_2$, overlays t, *J* 7.6, 3×O=C-C**H**$_2$), 2.43-2.49 (2H, m, ≡C-C**H**$_2$), 4.41 (6H, s, 3×C**H**$_2$-O-), 5.32 (6H, dt, *J* 5.6, 3.4, 3×-C**H**=C**H**-), 6.03 (1H, br s, N**H**); δ_C (100 MHz, $CDCl_3$) 14.3 (3×CH_2-**C**H_3), 14.9 (≡C-CH_2-**C**H_2), 22.8 (3×**C**H_2-CH_3), 25.0 (3×**C**H_2), 27.3 (3×=C-**C**H_2), 27.4 (3×**C**H_2-C=), 29.1 (3×**C**H_2), 29.3 (3×**C**H_2), 29.3 (6×**C**H_2), 29.9 (3×**C**H_2), 29.9 (3×**C**H_2), 31.9 (3×**C**H_2), 34.2 (3×**C**H_2), 36.0 (≡C-**C**H_2-CH_2), 58.7 (**C**R_3N), 62.7 (3×C-**C**H_2-O-), 69.6 (≡**C**H), 82.8 (HC≡**C**-), 129.9 (3×C=**C**) 130.2 (3×**C**=C), 171.2 (O=**C**), 173.6 (3×O=**C**); Accurate mass (ESI): Found: 910.7496 $C_{57}H_{100}O_7N$ (MH^+) requires 910.7494.

(*Z*)-2-((Oleoyloxy)methyl)-2-(pent-4-ynamido)propane-1,3-diyl dioleate (23)

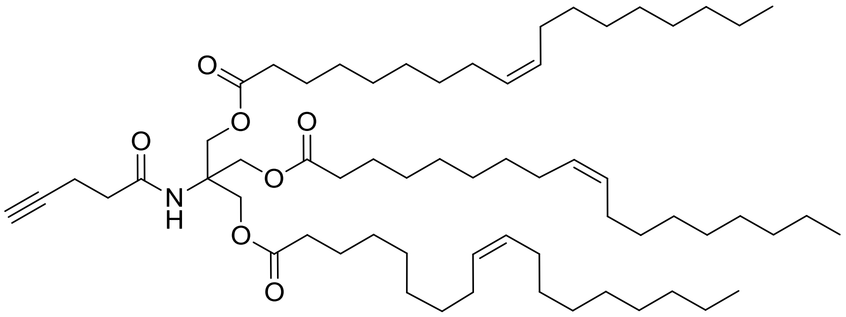

Synthesised in accordance with General Procedure B. Purification by column chromatography (5:1, petrol/ether) afforded the compound (501 mg, 63%) as a colourless, viscous oil; ν_{max}/cm^{-1} (thin film) 2922s, 2853s, 1742s, 1660s, 1545m, 1465m, 1378m, 1274m; δ_H (400 MHz, $CDCl_3$) 0.86 (9H, t, *J* 6.8, 3× CH_2-C**H_3**), 1.19-1.35 (60H, m, 30×C**H_2**) 1.54-1.64 (6H, m, 3×O=C-CH_2-C**H_2**), 1.94-2.03 (13H, m, 6×=CH-C**H_2** overlays ≡C**H**), 2.26-2.37 (8H, m, ≡C-CH_2-C**H_2**, overlays t, *J* 7.4, 3×O=C-C**H_2**), 2.43-2.49 (2H, m, ≡C-C**H_2**), 4.41 (6H, s, 3×C**H_2**-O-), 5.32 (6H, dt, *J* 5.7, 3.6, 3×-C**H**=C**H**-), 6.03 (1H, br s, N**H**); δ_C (100 MHz, $CDCl_3$) 14.3 (3×CH_2-**C**H_3), 14.9 (≡C-CH_2-**C**H_2), 22.8 (3×**C**H_2-CH_3), 25.0 (3×**C**H_2), 27.3 (3×=C-**C**H_2), 27.4 (3×**C**H_2-C=), 29.3 (3×**C**H_2), 29.3 (3×**C**H_2), 29.5 (3×**C**H_2), 29.7 (3×**C**H_2), 29.9 (3×**C**H_2), 29.9 (3×**C**H_2), 32.1 (3×**C**H_2), 34.2 (3×**C**H_2), 36.0 (≡C-**C**H_2-CH_2), 58.7 (**C**R_3N), 62.7 (3×C-**C**H_2-O-), 69.6 (≡**C**H), 82.8 (HC≡**C**-), 129.8 (3×C=**C**) 130.2 (3×**C**=C), 171.2 (O=**C**), 173.6 (3×O=**C**); Accurate mass (ESI): Found: 994.8415 $C_{63}H_{112}O_7N$ (MH^+) requires 994.8441.

(13Z,13'Z)-2-(((Z)-Docos-13-enoyloxy)methyl)-2-(pent-4-ynamido)propane-1,3-diyl bis(docos-13-enoate) (24)

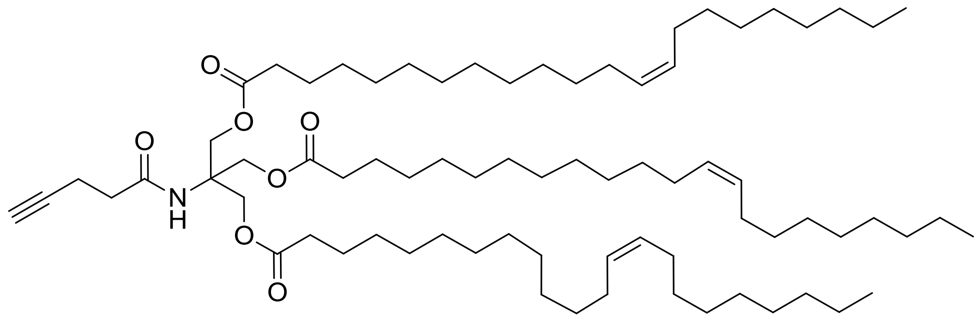

Synthesised in accordance with General Procedure B. Purification by column chromatography (5:1, petrol/ether) afforded the compound (648 mg, 70%) as a white solid; ν_{max}/cm^{-1} (thin film) 2921s, 2852s, 1743s, 1659s, 1545m, 1465m, 1378m; δ_H (400 MHz, $CDCl_3$) 0.86 (9H, t, *J* 6.7, 3× CH_2-C**H_3**), 1.19-1.35 (84H, m, 42×C**H_2**) 1.54-1.64 (6H, m, 3×O=C-CH_2-C**H_2**), 1.93-2.04 (13H, m, 6×=CH-C**H_2** and ≡C**H**), 2.27-2.38 (8H, m, ≡C-CH_2-C**H_2**, overlays t, *J* 7.6, 3×O=C-C**H_2**), 2.43-2.49 (2H, m, ≡C-C**H_2**), 4.41 (6H, s, 3×C**H_2**-O-), 5.32 (6H, dt, *J* 9.2, 4.6, 3×-C**H**=C**H**-), 6.03 (1H, br s, N**H**); δ_C (100 MHz, $CDCl_3$) 14.3 (3×CH_2-**C**H_3), 14.9 (≡C-CH_2-**C**H_2), 22.8 (3×**C**H_2-CH_3), 25.0 (3×**C**H_2), 27.4

(3×=C-**C**H_2), 29.3 (3×**C**H_2), 29.4 (3×**C**H_2), 29.5 (6×**C**H_2), 29.5 (3×**C**H_2), 29.7 (3×**C**H_2), 29.7 (3×**C**H_2), 29.7 (3×**C**H_2), 29.8 (3×**C**H_2), 29.9 (3×**C**H_2), 30.0 (3×**C**H_2), 32.1 (3×**C**H_2), 34.3 (3×**C**H_2), 36.0 (≡C-**C**H_2-CH_2), 58.7 (**C**R_3N), 62.7 (3×C-**C**H_2-O-), 69.5 (≡**C**H), 82.8 (HC≡**C**-), 130.0 (3×C=**C**) 130.1 (3×**C**=C), 171.20 (O=**C**), 173.6 (3×O=**C**); Accurate mass (ESI): Found: 1163.0320 $C_{75}H_{136}O_7N$ (MH+) requires 1163.0313.

Synthesis of sugar head groups

(2*S*,3*R*,4*S*,5*R*)-tetrahydro-2*H*-pyran-2,3,4,5-tetrayl tetraacetate (31)[2]

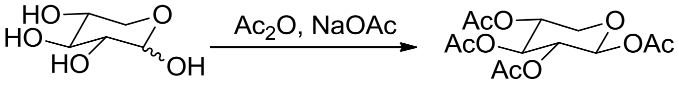

To a stirred suspension of anhydrous sodium acetate (4.36 g, 53.2 mmol) in acetic anhydride (25 mL) heated at reflux was added D(+)-xylose (2.0 g, 13.3 mmol). The reaction mixture was heated for 3 h before cooling to 100 °C and immediately transferred to an ice–water mixture and stirred vigorously until a gum formed. After decanting the aqueous portion, the gum was dissolved in DCM and washed successively with sat. aq. $NaHCO_3$ solution and brine. The organic layer was dried over $MgSO_4$ anhydrous and concentrated in vacuo. The title compound (4.05g, 97%) was obtained as a white solid and was used in subsequent reactions without further purification.

(2*R*,3*R*,4*S*,5*R*)-2-azidotetrahydro-2*H*-pyran-3,4,5-triyl triacetate (32)[3]

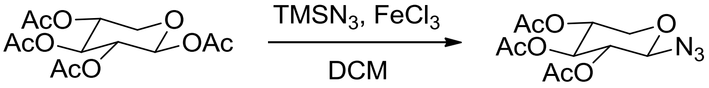

To a stirred solution of $FeCl_3$ (64 mg, 0.40 mmol) in dry DCM (10 mL) was added xylose tetraacetate **31** (4.18 g, 13.3 mmol) in DCM (20 mL). After stirring for 5 min, a solution of trimethylsilyl azide in DCM (10 mL) was added dropwise. The progress of reaction was monitored by thin layer chromatography (1:2 ethyl acetate/petrol). Once complete consumption of the starting material was observed, the reaction was quenched with sat. aq. $NaHCO_3$ solution. After separation, the aqueous layer was extracted with DCM and the combined organic extracts washed with brine, dried over

$MgSO_4$ and concentrated in vacuo to give the crude product. Purification by column chromatography (1:2 ethyl acetate/petrol benzene) afforded the title compound (2.29 g, 58%) as a colourless viscous oil. Data matched literature values.[3]

1-Azido-1-β-D-xylopranoside (27)[4]

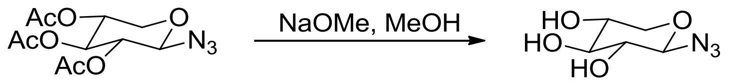

To a stirred solution of azide **32** (2.26g, 7.60 mmol) in methanol (20 mL) was added a catalytic amount of sodium methoxide (10 mg, 0.18 mmol). After stirring for 1 h the reaction mixture was neutralised with Amberlite IR-120[H^+] resin. The reaction mixture was filtered and concentrated in vacuo to afford the title compound (1.25g, 96%) as a white solid without the need for further purification; δ_H (400 MHz, MeOD) 3.09 (1H, app. t, *J* 8.4, C**H**OH-CHN_3), 3.22-3.31 (2H, m, COH-C**H**OH-COH & COH-C**H**OH-CH_2), 3.42-3.50 (1H, m, O-C**H**H'), 3.90 (1H, dd, *J* 10.6, 5.0, O-CH**H**'), 4.39 (1H, d, *J* 8.4, C**H**N_3); δ_C (100 MHz, $CDCl_3$) 67.8 (**C**H_2), 69.6 (**C**-OH), 73.5 (**C**-OH), 76.8 (**C**-OH), 91.4 (**C**-N_3).

Amphiphile synthesis and characterisation

General procedure

To each of 24 glass vials (18 mm × 45 mm) in a 4 × 6 array aluminium reaction block, was added a solution of azido sugar (1.0 equiv, ca. 15 mg) in 2:1 *t*-BuOH/water (1.5 mL). Alkyne (1.0 equiv) was added and the reaction block heated, with stirring, to 40 °C. After dissolution, copper powder (ca. 150 mg) was added and the reaction stirred for 24–48 h. The reaction mixture was cooled, diluted with ethanol (2 mL) and filtered through Celite® into 24 glass vials (25 mm × 75 mm). Concentration in vacuo on a Genevac EZ-2, followed by vacuum oven drying (50 °C, 3 h), afforded the amphiphile products.

MALDI–TOF results

Table S1: Double-chain amphiphiles.

Sugar	Double chain tail	Amphiphile	Predicted mass $[MNa]^+$	Experimental mass $[MNa]^+$
Glucose	C7	**33**	652.3	652.4
Glucose	C9	**34**	708.4	708.5
Glucose	C11	**35**	764.5	764.6
Glucose	C13	**36**	820.5	820.6
Glucose	Phyt	**37**	988.8	988.8
Glucose	Palm	**38**	872.6	872.7
Glucose	Ole	**39**	928.6	928.7
Glucose	Eruc	**40**	1040.7	1040.8
Glucose	Lin	**41**	924.7	924.7
Galactose	C7	**42**	652.3	652.1
Galactose	C9	**43**	708.4	708.3
Galactose	C11	**44**	764.5	764.4
Galactose	C13	**45**	820.5	820.6
Galactose	C15	**46**	876.6	876.5
Galactose	C17	**47**	932.7	932.6
Galactose	Phyt	**48**	988.7	988.7
Galactose	Palm	**49**	872.6	872.5
Galactose	Ole	**50**	928.6	928.6
Galactose	Eruc	**51**	1040.7	1040.7
Galactose	Lin	**52**	924.6	924.6
Xylose	C7	**53**	622.3	622.3
Xylose	C9	**54**	678.4	678.5
Xylose	C11	**55**	734.5	734.6
Xylose	C13	**56**	790.5	790.7
Xylose	C15	**57**	846.6	846.7
Xylose	C17	**58**	902.6	902.8
Xylose	Phyt	**59**	958.8	958.8
Xylose	Palm	**60**	842.5	842.7
Xylose	Ole	**61**	898.6	898.7

Xylose	Eruc	**62**	1010.7	1010.9
Xylose	Lin	**63**	894.7	894.7
Mannose	C7	**64**	652.3	652.2
Mannose	C9	**65**	708.4	708.3
Mannose	C11	**66**	764.5	764.4
Mannose	Phyt	**67**	988.7	988.7
Mannose	Palm	**68**	872.6	872.5
Mannose	Ole	**69**	928.6	928.6
Lactose	C7	**70**	814.1	814.3
Lactose	C9	**71**	870.5	870.4
Lactose	C11	**72**	926.6	926.5
Lactose	C13	**73**	982.6	982.5
Lactose	Phyt	**74**	1150.8	1150.8
Lactose	Palm	**75**	1034.6	1034.6
Lactose	Ole	**76**	1090.7	1090.7
Lactose	Eruc	**77**	1202.8	1203.5
Lactose	Lin	**78**	1086.1	Not found

Table S2: Triple chain amphiphiles.

Sugar	Double chain tail	Amphiphile	Predicted mass $[MNa]^+$	Experimental mass $[MNa]^+$
Glucose	C7	**79**	807.5	807.6
Glucose	C9	**80**	891.6	891.7
Glucose	C11	**81**	975.7	975.7
Glucose	Phyt	**82**	1312.0	1312.0
Glucose	Palm	**83**	1137.8	1137.8
Glucose	Ole	**84**	1221.9	1221.9
Glucose	Eruc	**85**	1390.0	1390.0
Galactose	C7	**86**	807.5	807.4
Galactose	C9	**87**	891.6	891.5
Galactose	C11	**88**	975.7	975.7
Galactose	Phyt	**89**	1312.0	1312.1
Galactose	Palm	**90**	1137.8	1137.8
Galactose	Ole	**91**	1221.9	1221.9
Galactose	Eruc	**92**	1390.1	1390.1
Xylose	C7	**93**	777.5	777.6
Xylose	C9	**94**	861.6	861.7
Xylose	C11	**95**	923.3	945.6
Xylose	Phyt	**96**	1282.0	1282.1
Xylose	Palm	**97**	1107.8	1107.9
Xylose	Ole	**98**	1191.9	1192.0
Xylose	Eruc	**99**	1360.0	1360.0
Mannose	C7	**100**	807.5	807.4
Mannose	C9	**101**	891.6	891.5
Mannose	C11	**102**	975.7	975.7
Mannose	Phyt	**103**	1312.0	1312.1
Mannose	Palm	**104**	1137.8	1137.9
Mannose	Ole	**105**	1221.9	1221.9
Lactose	C7	**106**	969.5	969.5
Lactose	C9	**107**	1053.6	1053.6
Lactose	C11	**108**	1137.7	1137.7
Lactose	Phyt	**109**	1474.1	1474.1
Lactose	Palm	**110**	1299.9	Not found

Lactose	Ole	**111**	1384.0	1383.9
Lactose	Eruc	**112**	1552.1	Not found

^{13}C NMR results

A selection of 15 amphiphiles (3 for each sugar) were randomly selected for ^{13}C NMR spectroscopy and cover a range of different double and triple chain tails.

Glucose – 2 × Ole (**39)**

δ_C (100 MHz, CD_3OD) 13.1 (×2), 16.7, 22.3 (×2), 24.6 (×2), 26.7 (×4), 28.7 (×2), 28.8 (×2), 28.9 (×2), 29.0 (×2), 29.1 (×2), 29.2 (×2), 29.4 (×2), 29.5 (×2), 31.7 (×2), 33.4 (×2), 46.2, 57.4, 61.0, 64.8 (×2), 69.5, 72.6, 77.1, 79.8, 88.2, 123.9, 129.4 (×2), 129.5 (×2), 142.2, 172.5, 173.3 (×2).

Galactose – 2 × C13 (**45)**

δ_C (100 MHz, CD_3OD) 13.1 (×2), 16.7, 22.3 (×2), 24.6 (×2), 28.8 (×2), 29.0 (×2), 29.1 (×2), 29.2 (×2), 29.4 (×2), 29.4 (×2), 29.4 (×2), 29.4 (×2), 31.7 (×2), 33.4 (×2), 46.2, 57.5, 60.9, 64.8 (×2), 68.9, 70.1, 73.9, 78.6, 88.9, 123.4, 142.4, 172.5, 173.4 (×2).

Galactose – 2 × Palm (**49)**

δ_C (100 MHz, CD_3OD) 13.1 (×2), 16.7, 22.3 (×2), 24.6 (×2), 26.7 (×2), 26.8 (×2), 28.7 (×2), 28.7 (×2), 28.8 (×2), 28.9 (×2), 29.4 (×2), 29.4 (×2), 31.5 (×2), 33.4 (×2), 46.2, 57.5, 60.9, 64.8 (×2), 68.9, 70.1, 73.6, 78.6, 88.9, 123.4, 129.4 (×2), 129.5 (×2), 142.4, 172.5, 173.4 (×2).

Xylose – 2 × C15 (**57)**

δ_C (100 MHz, CD_3OD) 13.1 (×2), 16.7, 22.3 (×2), 24.6 (×2), 28.8 (×2), 29.0 (×2), 29.1 (×2), 29.2 (×2), 29.4 (×2), 29.4 (×4), 29.4 (×4), 29.7 (×2), 31.7 (×2), 33.4 (×2), 46.2, 57.4, 64.9 (×2), 68.5, 69.2, 72.5, 77.2, 88.9, 123.8, 142.2, 172.6, 173.3 (×2).

Mannose – 2 × C7 (**64)**

δ_C (100 MHz, CD_3OD) 13.0 (×2), 16.6, 22.3 (×2), 24.6 (×2), 28.7 (×2), 28.7 (×2), 31.5 (×2), 33.4 (×2), 46.2, 57.4, 61.1, 64.8 (×2), 67.2, 68.7, 71.2, 77.4, 87.0, 124.6, 142.5, 172.5, 173.3 (×2).

Mannose – 2 × Phyt (**67**)

δ_C (100 MHz, CD_3OD) 16.8, 18.7, 18.7, 18.8 (×2), 18.8, 18.8, 21.6 (×2), 21.7 (×2), 24.0, 24.1, 24.1 (×2), 24.5, 24.5, 27.8 (×2), 30.1, 30.1, 32.5 (×2), 32.6 (×2), 36.5, 36.6, 36.8, 36.8, 36.9, 36.9, 37.0, 37.1, 37.1, 37.2, 39.2 (×2), 41.0, 41.1, 46.1, 57.4, 61.1, 64.8 (×2), 67.2, 68.7, 71.2, 77.3, 87.0, 124.5, 142.5, 172.5, 172.7 (×2).

Lactose – 2 × C9 (**71**)

δ_C (100 MHz, CD_3OD) 13.0 (×2), 16.6, 22.3 (×2), 25.6 (×2), 28.8 (×2), 29.0 (×4), 29.2 (×2), 31.7 (×2), 33.4 (×2), 46.2, 57.4, 60.1, 61.1, 64.8 (×2), 68.9, 71.1, 72.2, 73.4, 75.5, 75.8, 78.2, 78.2, 88.0, 103.7, 123.9, 142.2, 172.5, 173.4 (×2).

Lactose – 2 × Eruc (**77**)

δ_C (100 MHz, CD_3OD) 13.1 (×2), 16.7, 22.4 (×2), 24.6 (×2), 26.8 (×4), 28.7 (×2), 28.8 (×4), 28.9 (×2), 29.0 (×2), 29.1 (×4), 29.2 (×4), 29.4 (×4), 29.5 (×2), 31.7 (×2), 33.4 (×2), 46.2, 57.4, 60.1, 61.1, 64.8 (×2), 68.9, 71.1, 72.2, 73.4, 75.5, 75.8, 78.2, 78.2, 88.0, 103.7, 123.9, 129.4 (×2), 129.5 (×2), 142.2, 172.5, 173.3 (×2).

Glucose – 3 × C9 (**80**)

δ_C (100 MHz, CD_3OD) 13.1 (×3), 21.0, 22.3 (×3), 24.6 (×3), 28.8 (×3), 29.0 (×3), 29.1 (×3), 29.2 (×3), 31.7 (×3), 33.5 (×3), 35.0, 57.7, 61.0, 61.6 (×3), 69.5, 72.6, 77.1, 79.7, 88.2, 121.3, 146.2, 173.3 (×3), 173.5.

Glucose – 3 × Palm (**83**)

δ_C (100 MHz, CD_3OD) 13.0 (×3), 21.1, 22.3 (×3), 24.6 (×3), 28.7 (×3), 28.7 (×3), 28.8 (×3), 28.8 (×3), 29.4 (×3), 29.4 (×3), 31.5 (×3), 33.4 (×3), 35.4, 60.6, 61.0 (×3), 62.3, 69.5, 72.6, 77.1, 79.7, 88.2, 121.4, 129.4 (×3), 129.5 (×3), 146.2, 174.2, 174.6 (×3).

Galactose – 3 × Phyt (**89**)

δ_C (100 MHz, CD_3OD) 18.7, 18.8, 18.9, 21.0, 21.7 (×3), 21.8 (×3), 24.1, 24.1, 24.1 (×3), 24.2, 24.5 (×3), 27.8 (×3), 30.1 (×3), 32.5, 32.5 (×3), 32.6 (×2), 35.1, 36.6 (×3), 36.7 (×3), 36.8, 36.9, 36.9, 37.0, 37.0 (×3), 37.1 (×3), 37.1 (×3), 37.2, 37.2, 39.2 (×3), 41.1 (×2), 41.1, 57.6, 61.0, 61.6 (×3), 68.9, 70.0, 73.9, 78.5, 88.8, 120.8, 146.3, 172.6 (×3), 173.5.

Xylose – 3 × C11 (**95**)

δ_C (100 MHz, CD_3OD) 13.2 (×3), 21.3, 22.5 (×3), 24.8 (×3), 29.0 (×3), 29.1 (×3), 29.2 (×3), 29.3 (×3), 29.5 (×6), 31.8 (×3), 33.6 (×3), 35.5, 61.2 (×3), 62.4, 68.6, 69.4, 72.6, 77.3, 88.9, 121.3, 146.4, 174.4, 174.8 (×3).

Xylose – 3 × Eruc (**99**)

δ_C (100 MHz, CD_3OD) 13.0 (×3), 21.1, 22.3 (×3), 24.6 (×3), 26.7 (×3), 26.7 (×3), 28.8 (×3), 28.9 (×3), 28.9 (×3), 29.0 (×3), 29.1 (×3), 29.2 (×3), 29.2 (×3), 29.2 (×3), 29.3 (×3), 29.3 (×3), 29.4 (×3), 29.4 (×3), 31.7 (×3), 33.4 (×3), 35.3, 61.0 (×3), 62.3, 68.4, 69.3, 72.5, 77.2, 88.8, 121.2, 129.4 (×3), 129.5 (×3), 146.3, 174.2, 176.6 (×3).

Mannose – 3 × Ole (**105**)

δ_C (100 MHz, CD_3OD) 13.0 (×3), 21.0, 22.3 (×3), 24.6 (×3), 26.7 (×3), 26.7 (×3), 28.7 (×3), 28.8 (×3), 28.8 (×3), 28.9 (×3), 29.0 (×3), 29.2 (×3), 29.4 (×3), 29.4 (×3), 31.7 (×3), 33.4 (×3), 35.3, 61.0 (×3), 61.2, 62.3, 67.2, 68.7, 71.1, 77.1, 86.9, 122.1, 129.4 (×3), 129.5 (×3), 146.5, 174.2, 174.6 (×3).

Lactose – 3 × C7 (**106**)

δ_C (100 MHz, CD_3OD) 13.0 (×3), 21.0, 22.2 (×3), 24.6 (×3), 28.7 (×3), 28.1 (×3), 31.4 (×3), 33.4 (×3), 35.2, 60.1, 60.6, 61.0, 61.1 (×3), 61.7, 71.1, 72.2, 73.4, 75.4, 75.8, 78.1, 78.3, 87.9, 103.7, 121.3, 146.3, 173.5 (×3), 174.7.

^{13}C NMR spectra

Glucose - 2 x Oleic
Amphipihle 39

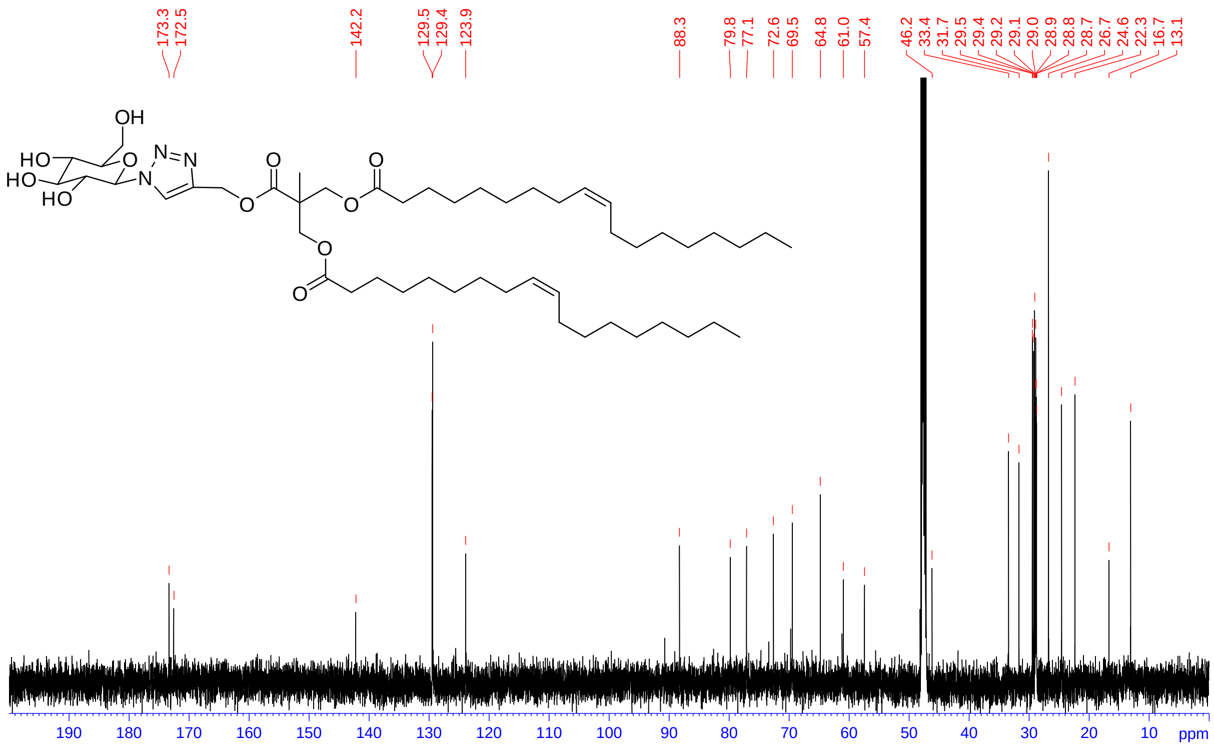

Galactose - 2 x C13
Amphiphile 45

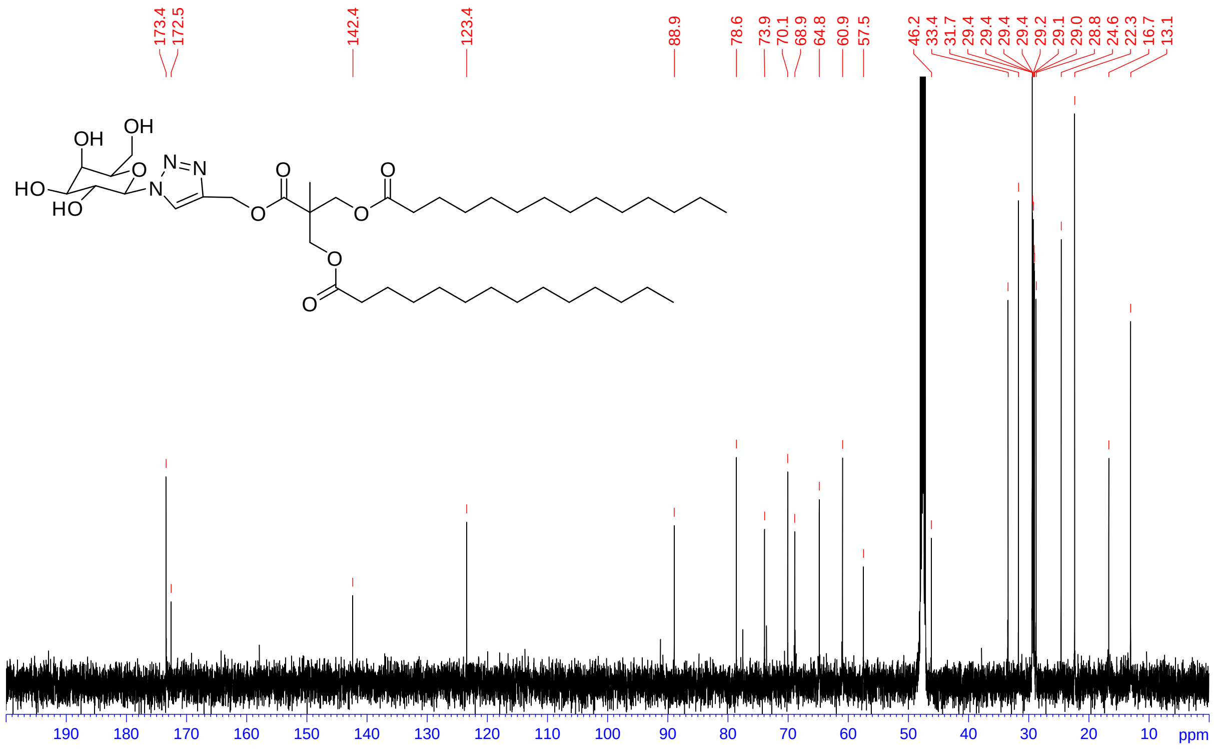

Galactose - 2 x Palmitoleic
Amphiphile 49

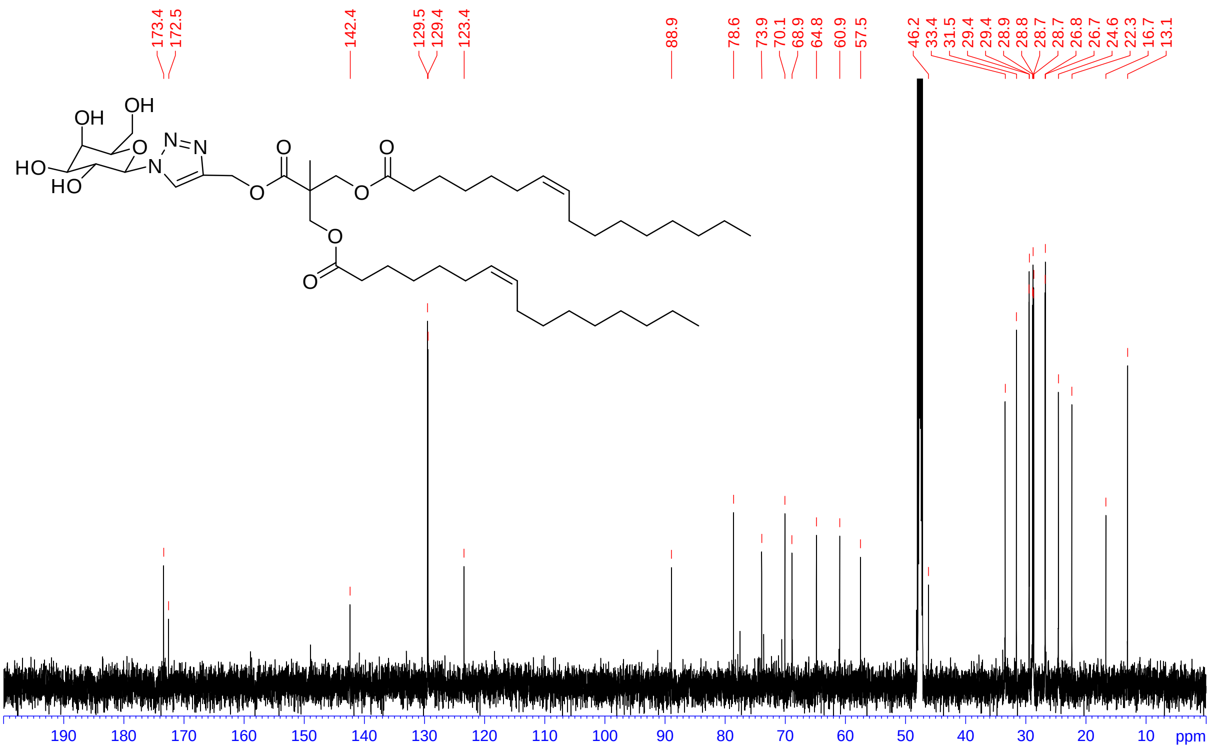

Xylose - 2 x C15
Amphiphile 57

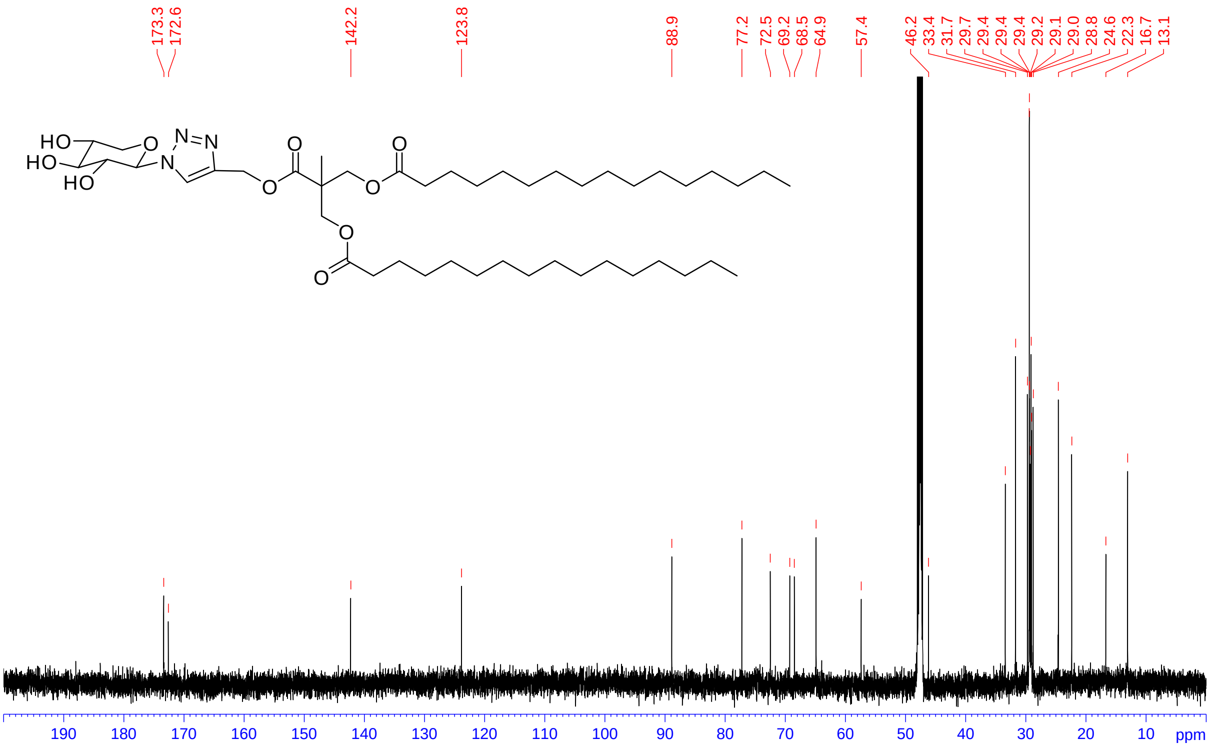

Mannose - 2 x C7
Amphiphile 64

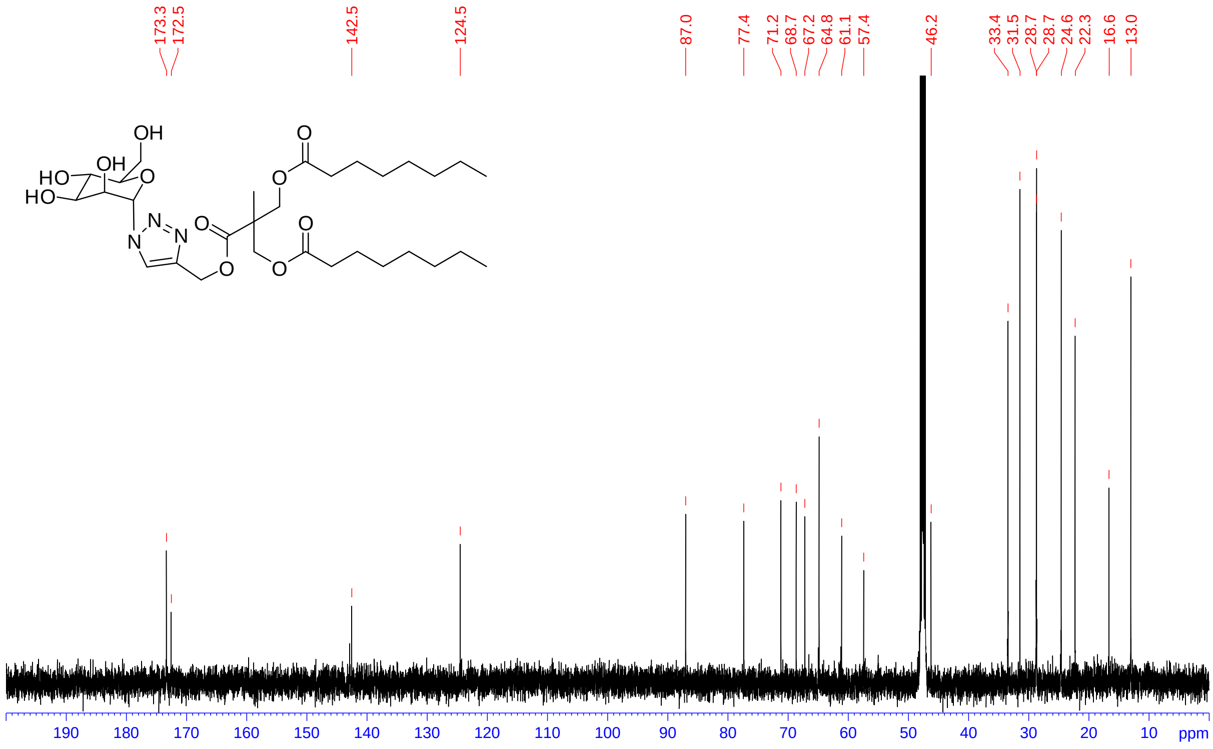

Mannose - 2 x Phytanic
Amphiphile 67

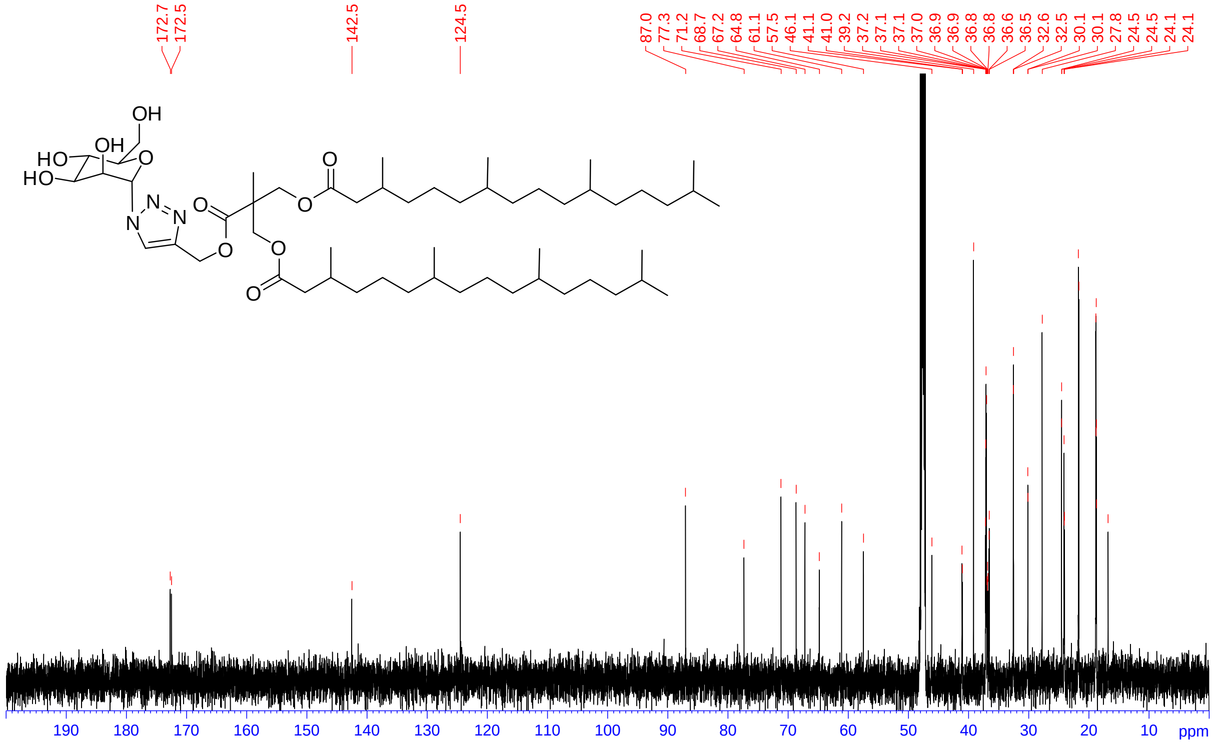

Lactose - 2 x C9
Amphiphile 71

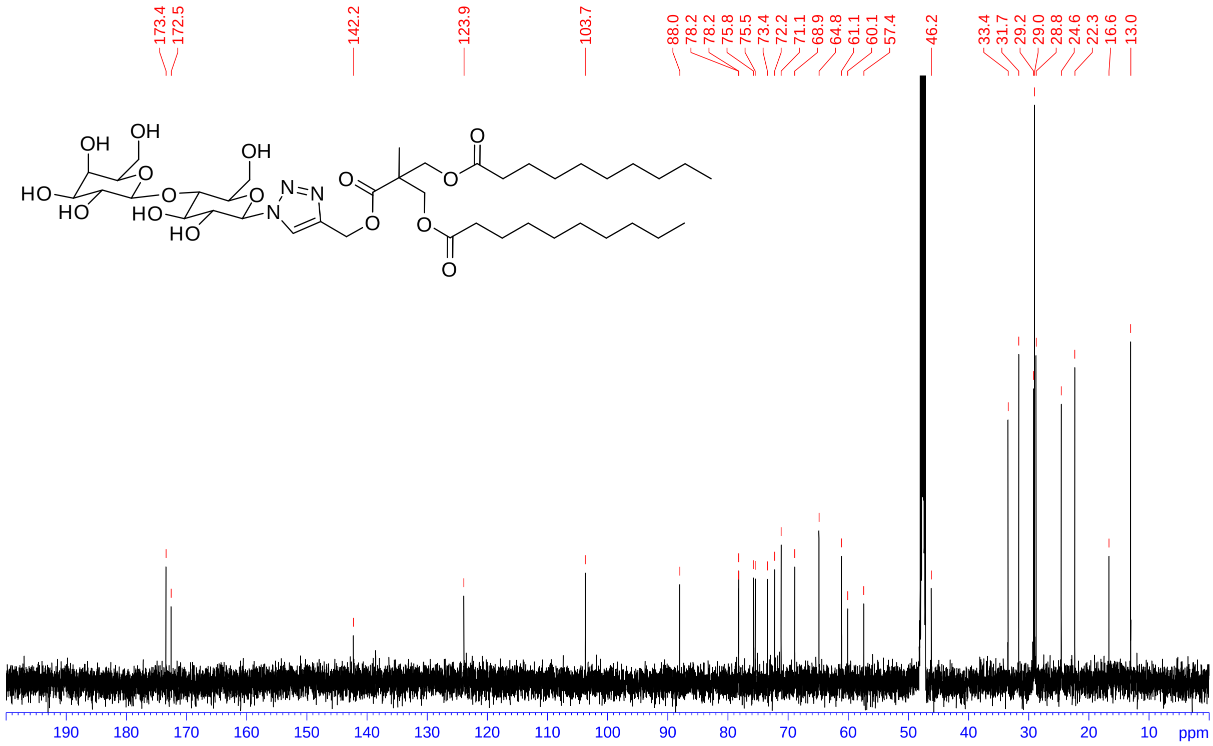

Lactose - 2 x Erucic
Amphiphile 77

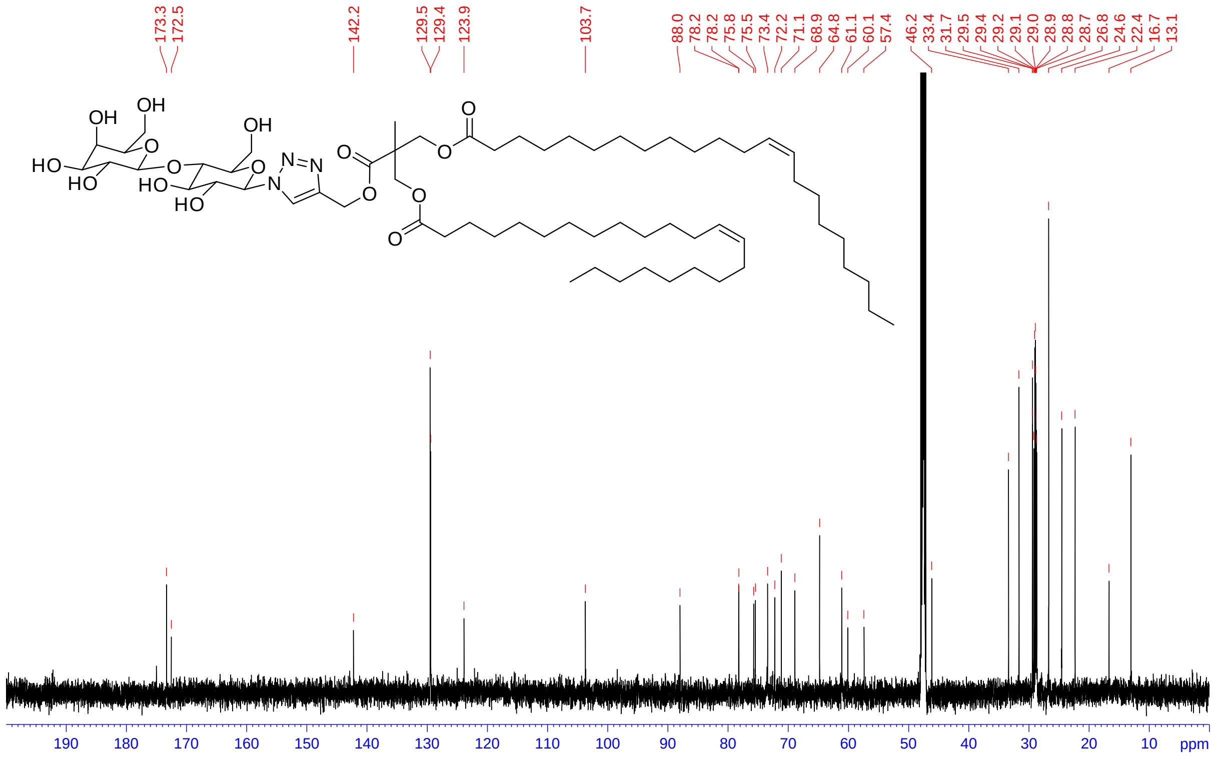

Glucose - 3 x C9
Amphiphile 80

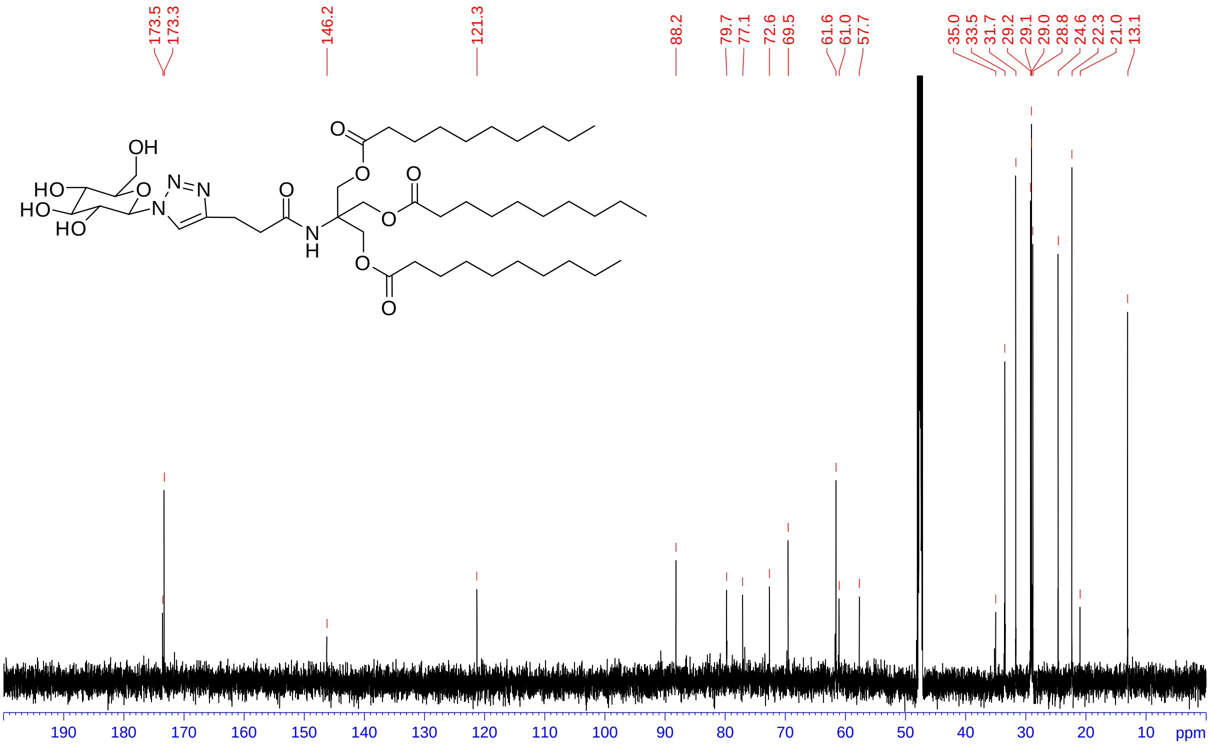

Glucose - 3 x Palmitoleic
Amphiphile 83

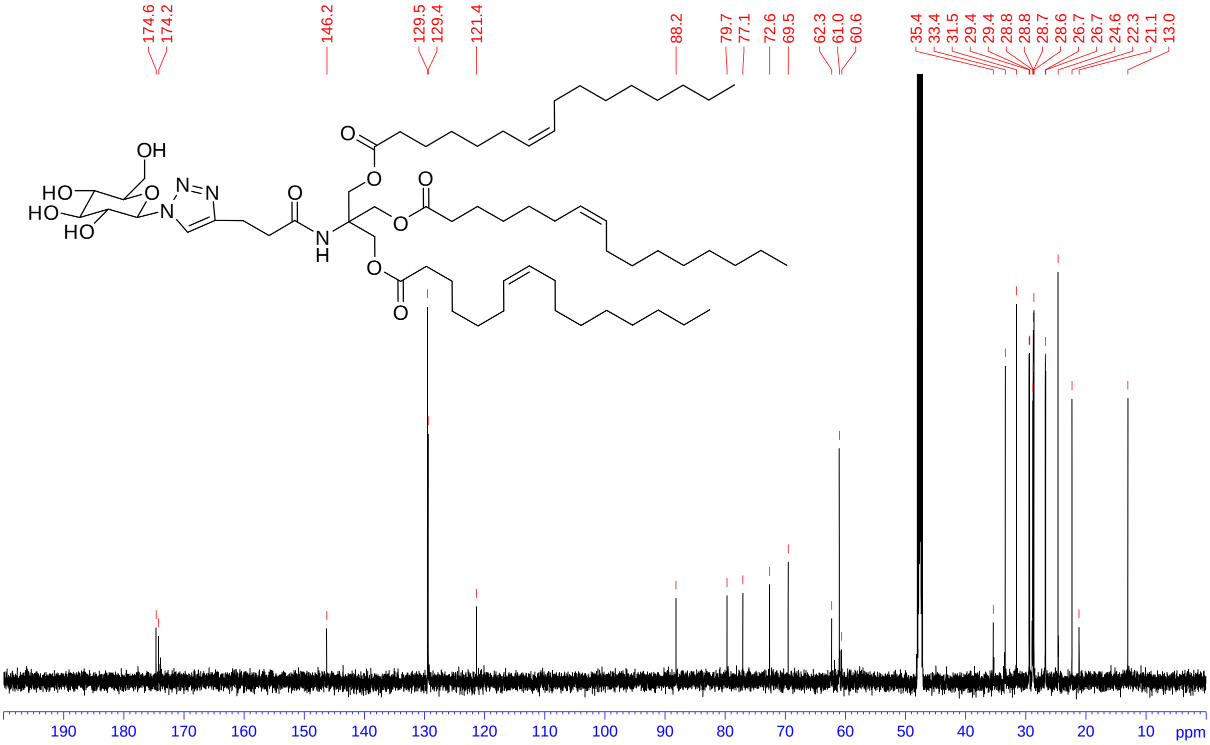

Galactose - 3 x Phytanic
Amphiphile 89

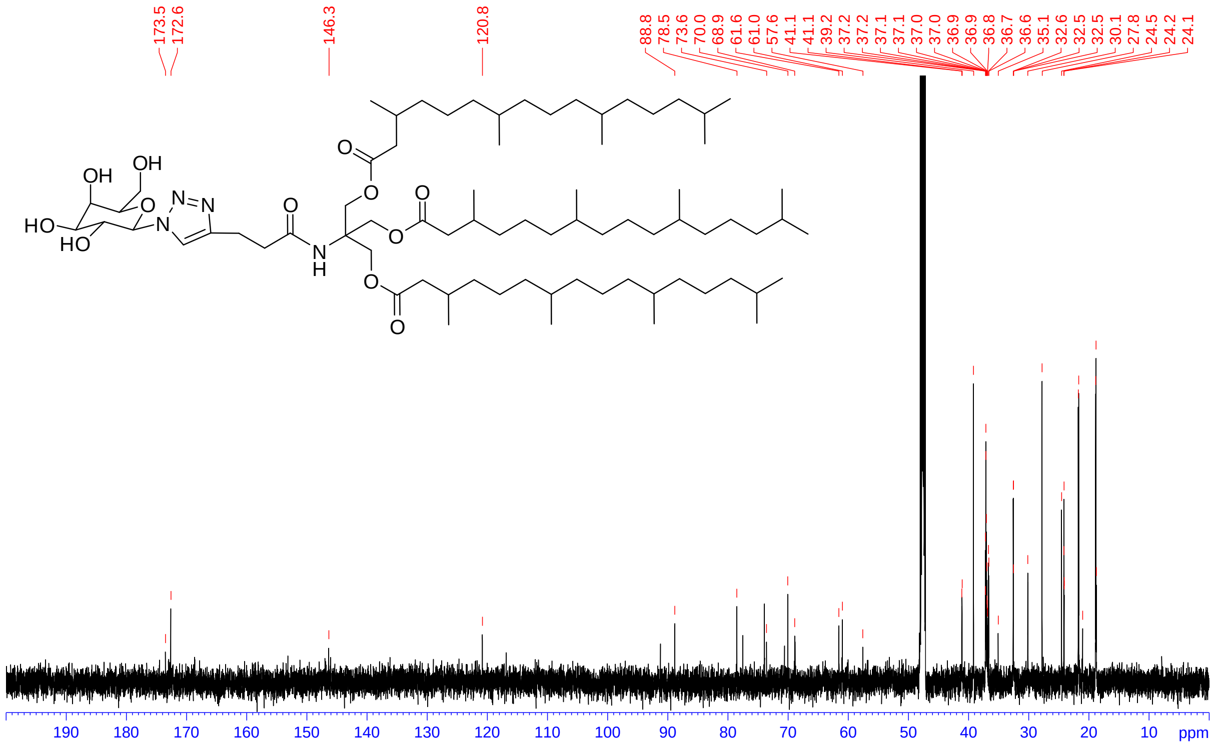

Xylose - 3 x C11
Amphiphile 95

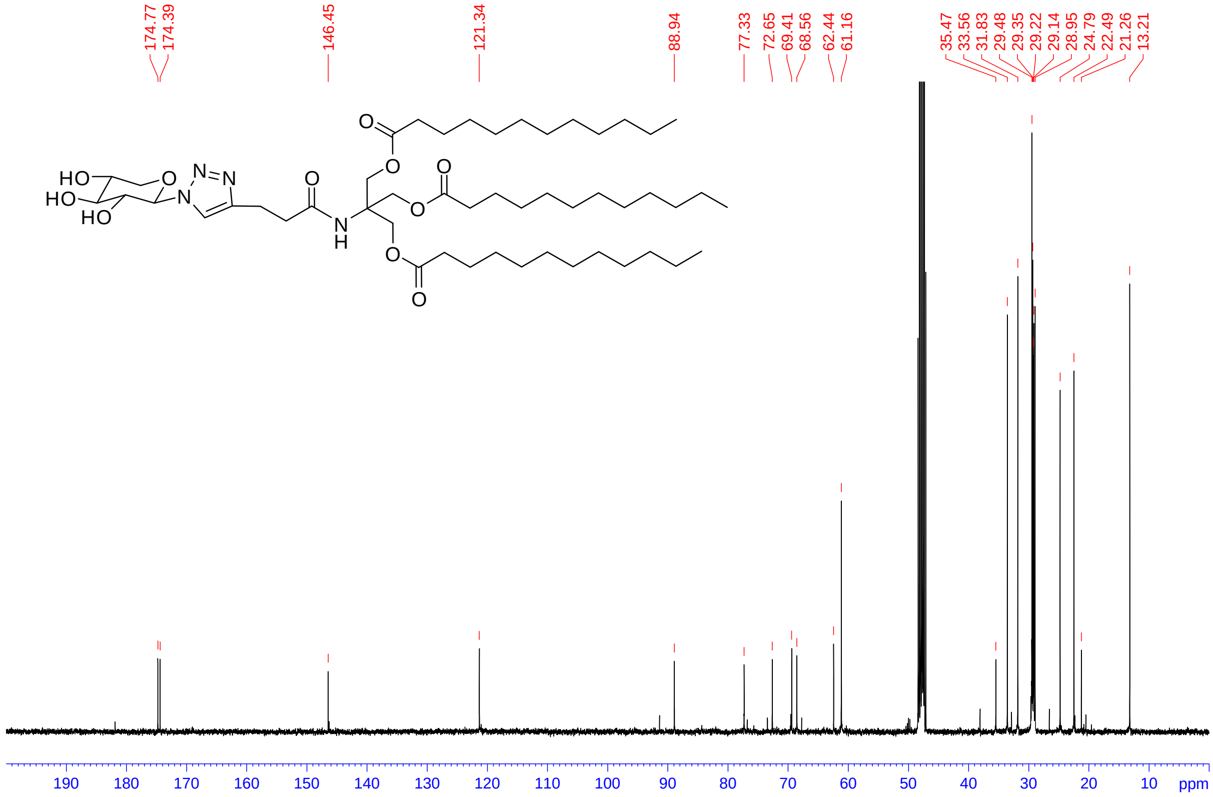

Xylose - 3 x Erucic
Amphiphile 99

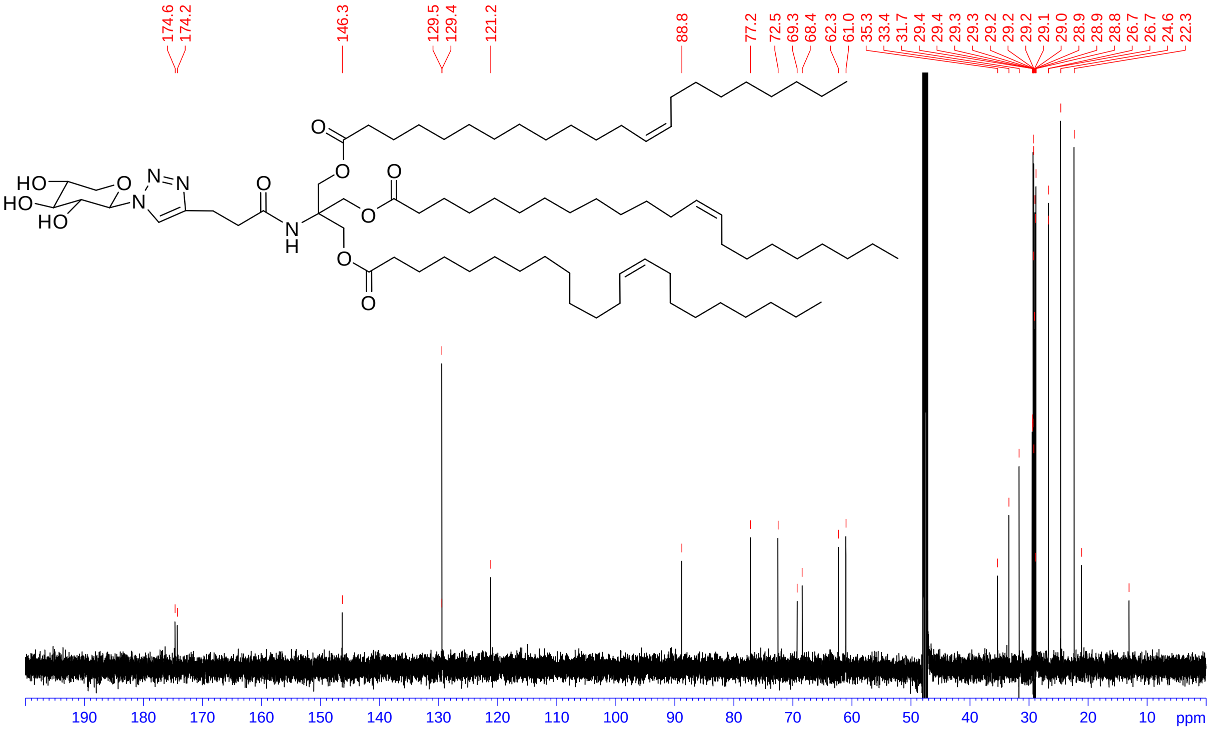

Mannose - 3 x Oleic
Amphiphile 105

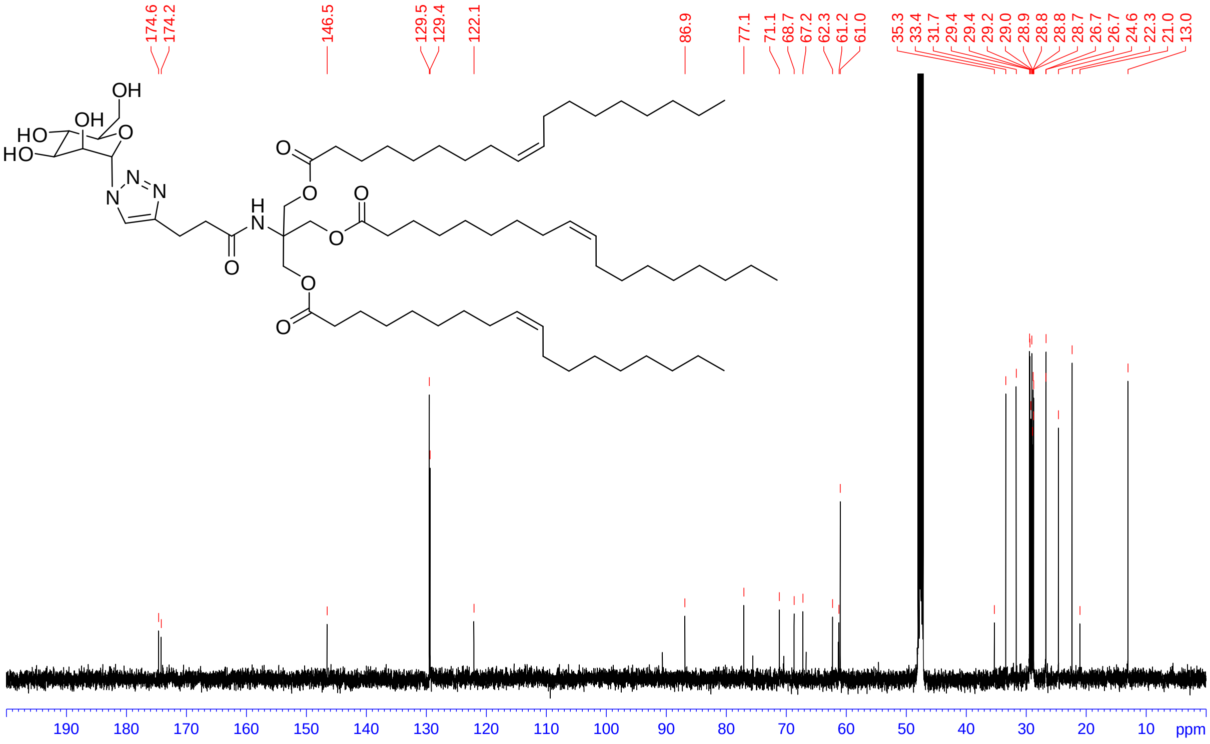

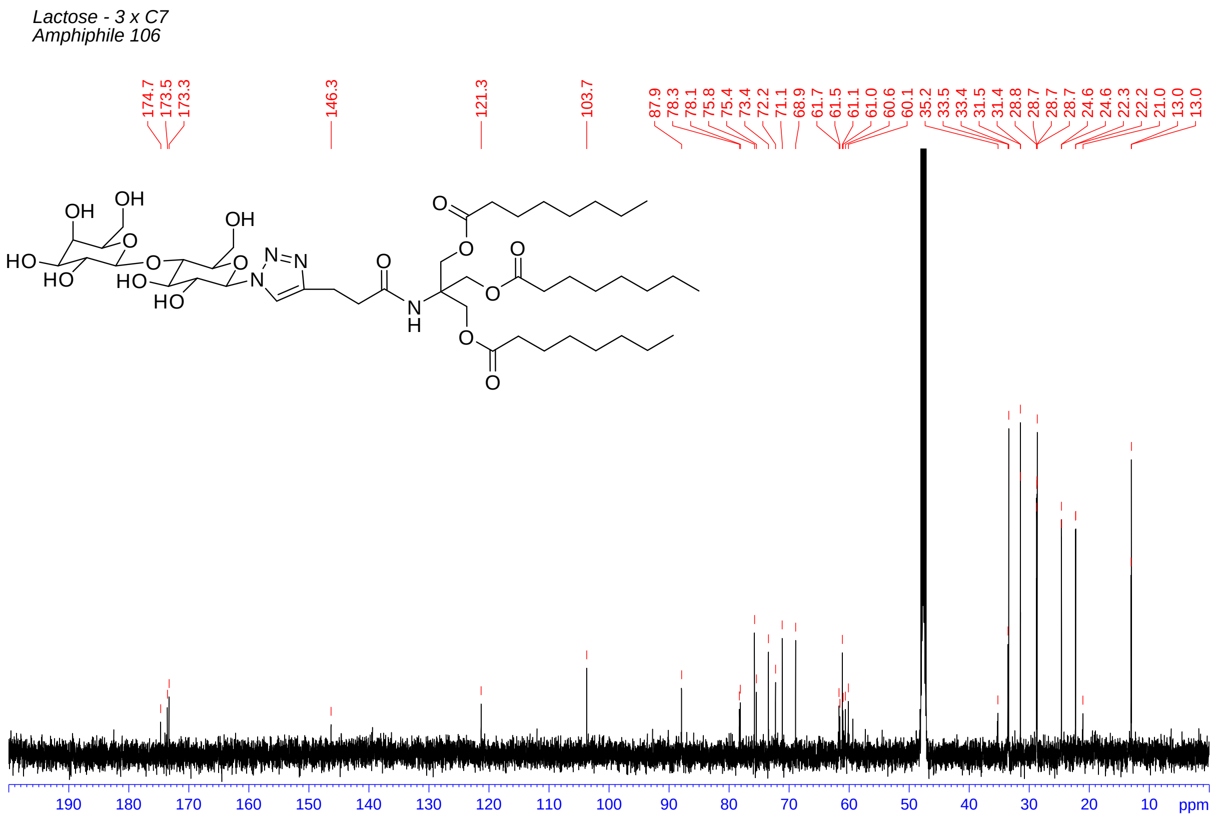

Preliminary SAXS analysis

The internal liquid crystalline structure was determined using small-angle X-ray scattering. Each amphiphile was dispensed into a 96-well plate and an excess of water (70% w/v) was added to each well using a Mosquito® liquid dispensing robot. The samples were equilibrated under controlled conditions of temperature and humidity for 5 days before SAXS analysis was carried out. Samples were analysed within the 96-well plate using a bespoke sample holder at the SAXS beamline at the Australian Synchrotron. The resulting diffraction patterns were analysed using the IDL-based AXcess software package[5] and the number and distribution of reflections used to assign the mesophase.

Table S3: SAXS data for compounds **33**, **53** and **70** at 70% w/v at 25 °C.

Amphiphile		Phase	Lattice parameter (Å)
Glucose – 2 × C7	**33**	IA3D	164.8
Xylose – 2 × C7	**53**	Micellar	n/a
Lactose – 2 × C7	**70**	Lamellar	56.2

References

1. V. Truong, I. Blakey, A. K. Whittaker, *Biomacromolecules* **2012**, *13*, 4012-4021.

2. J. r. m. Camponovo, C. Hadad, J. Ruiz, E. Cloutet, S. Gatard, J. Muzart, S. Bouquillon, D. Astruc, *J. Org. Chem.* **2009**, *74*, 5071-5074.

3. S. B. Salunke, N. S. Babu, C.-T. Chen, *Chem. Commun.* **2011**, *47*, 10440-10442.

4. T. Bravman, V. Belakhov, D. Solomon, G. Shoham, B. Henrissat, T. Baasov, Y. Shoham, *J. Biol. Chem.* **2003**, *278*, 26742-26749.

5. J. M. Seddon, A. M. Squires, C. E. Conn, O. Ces, A. J. Heron, X. Mulet, G. C. Shearman, R. H. Templer, *Philos. Trans. R. Soc., A* **2006**, *364*, 2635-2655.
